# Parents’ perception of enhanced oncofertility care in childhood cancer patients following routine fertility informing

**DOI:** 10.3389/fped.2026.1925381

**Published:** 2026-10-21

**Authors:** Egle Stukaite-Ruibiene, Nikita H. Z. Clasen, Marry M. van den Heuvel-Eibrink, Jelena Rascon

**Affiliations:** 1 Center for Pediatric Oncology and Hematology, Vilnius University Hospital Santaros Klinikos, Vilnius, Lithuania; 2 Department of Anatomy, Histology and Anthropology, Faculty of Medicine, Vilnius University, Vilnius, Lithuania; 3 Princess Máxima Center for Pediatric Oncology, Utrecht, Netherlands; 4 Division of Child Health, Wilhelmina Children’s Hospital, and University of Utrecht, Utrecht, Netherlands; 5 Faculty of Medicine, Vilnius University, Vilnius, Lithuania

**Keywords:** childhood cancer, fertility, late effects, oncology, pediatrics, survivors

## Abstract

**Background:**

The 5-year survival rate of childhood cancer is >80%, indicating a large number of survivors. Infertility is a late effect of cancer treatment. This study aimed to evaluate the perception of early routine fertility informing of families of children with cancer as part of oncofertility care before and after the implementation of routine informing procedure.

**Methods:**

We evaluated and compared the quality of oncofertility care before and after routine fertility informing was implemented. The baseline group was evaluated between 1 July 2021 and 1 July 2022, using the oncofertility care evaluation questionnaire. Infertility risk was determined retrospectively in this group. On 1 July 2022, routine fertility informing was implemented. Thereafter, infertility risk was assessed prospectively, and families were informed by a dedicated staff member. The second group completed the same questionnaire.

**Results:**

Of the respondents in the second group, 63.6% reported having had an opportunity to discuss fertility (vs. 37.5% of the first group; *p* = 0.002), 42.4% had received supportive material (vs. 10.7%; *p* = 0.003), 24.2% had questions about fertility after education (vs. 42.9%; *p* = 0.003), and 59% felt knowledgeable about the effects of treatment on fertility (vs. 32.1%, *p* = 0.001). Infertility risk was estimated correctly by 15.2% of respondents before implementation of routine informing compared with 63.6% after implementation.

**Conclusions:**

Routine and early fertility informing of families of children with cancer as standard of care significantly improved patients' perception of oncofertility care. Parental awareness of fertility-related issues increased substantially.

## Introduction

Cancer treatment has multiple long-term effects on survivors later in life (1, 2). This is particularly relevant for patients who are childhood cancer survivors (CCSs) because the 5-year survival rate of childhood cancer exceeds 80% (3, 4). Gonadal damage is one of the late effects reported to be of importance to CCSs and their parents. It can result in hormonal insufficiencies, impaired fertility, or both (5–7). In female patients, high cumulative doses of alkylating agents are particularly associated with gonadal toxicity, as supported by current clinical practice guidelines (8). Abdominal surgery, ovarian surgery, and abdominal radiotherapy can result in primary ovarian insufficiency, follicular atresia, and premature menopause (9, 10). Cranial irradiation can result in hypogonadotropic hypogonadism (7, 11, 12). Female CCSs may have more difficulty becoming pregnant than their healthy siblings, and the risks of obstetric complications, gonadal damage, subsequent infertility, and premature ovarian insufficiency are higher among female CCSs than among the general population (5, 10, 13–15). In male patients, treatment with alkylating agents, testicular or cranial radiotherapy, and orchiectomy can result in impaired spermatogenesis, testosterone deficiency, and sexual dysfunction (5, 16). Fewer male CCSs have been reported to have biological children than their siblings (17).

Late sequelae of childhood cancer treatment, including infertility, are associated with impaired long-term quality of life (18, 19). Appropriate fertility counseling for patients and their families, in relation to the child's age and developmental level, is beneficial irrespective of decisions regarding fertility preservation (20, 21). Providing information on fertility and other late treatment effects can improve the quality of life of CCSs (22). The International Late Effects of Childhood Cancer Guideline Harmonization Group (IGHG) recommends educating all CCSs about fertility damage, irrespective of the infertility risk, and offering fertility preservation before treatment (16, 23). Other influential international guidelines, such as those from the American Society of Clinical Oncology (24) and the European Society for Medical Oncology (25), support routine fertility counseling for all pediatric patients with cancer. However, studies have shown that many CCSs reported that fertility counseling at diagnosis was insufficient and that they lacked information about reproductive health and their fertility status (26, 27). One study showed that 80.2% of adolescents with cancer reported having received information about the risk of infertility before treatment; however, only 52.3% stated that they felt sufficiently informed (28). Some adolescents and young adults who survived cancer later regret their decisions regarding fertility preservation; thus, the education of patients and parents regarding fertility must be improved (29). Our previous study showed that adult CCSs were not properly informed of their infertility risk upfront at the time of cancer treatment, and their overall understanding of treatment-related fertility issues was limited (30). Therefore, appropriate information and counseling must be provided early in treatment.

Until recently, tools dedicated to the evaluation of infertility risk in children treated for cancer were unavailable. Therefore, a gonadal damage risk stratification tool (triage) was developed at the Princess Máxima Center (Utrecht, the Netherlands; hereinafter, “Máxima”) (31) to aid in the evaluation of gonadal damage risk (low or high) according to the IGHG guidelines (16, 23). An older tool, the cyclophosphamide equivalent dose (CED) (32), was used to evaluate the risk of fertility impairment caused by all cancer treatment protocols (31). In addition, there were no validated tools to evaluate the quality of fertility information and counseling. Accordingly, in collaboration with Máxima, we developed, translated, and adapted an oncofertility care evaluation questionnaire (OCEQ) (22). In our recent study, we amended the gonadal damage risk stratification tool and adapted it to Lithuanian healthcare: We calculated CEDs for all treatment protocols used at Vilnius University Hospital Santaros Klinikos (VUHSK), Vilnius, Lithuania, because some of them differed from those used at Máxima (33). Of the 120 parents of patients, 56 (46.7%) completed the questionnaires and reported that information on fertility damage was insufficient, as it was provided along with other effects of cancer treatment (33). We concluded that routine fertility information and documentation, with stratification of patients with low and high risk of infertility, are crucial before or early in cancer treatment and should be implemented in clinical practice. The results suggested the need for dedicated personnel to coordinate fertility care, as pioneered at Máxima (31). Subsequently, a strategy for providing routine oncofertility information was initiated at our institution on 1 July 2022.

In this study, we aimed to evaluate whether the prospective provision of standard-of-care fertility information to all families with children diagnosed with cancer at VUHSK improved the perceptions of the quality of oncofertility care and knowledge. We compared these perceptions with those reported before the implementation of routine fertility informing (33).

## Material and methods

### Questionnaire evaluating fertility care

The OCEQ was developed and adapted to the VUHSK setting as a part of the Horizon-2020 TREL project (22). Responses to the provided statements were expressed on a 5-point Likert scale (from “Completely disagree” to “Fully agree”). The OCEQ included various suitable questions from validated questionnaires administered in adult settings: the Decision Regret Scale (34), the Decisional Conflict Scale (35), the Dutch Reproductive Concern Scale (36), and the Patient-Centeredness Questionnaire–Infertility (originally developed for subfertile couples) (37). Some questions were amended slightly for the pediatric oncology setting, and several new questions were added.

To facilitate the effective use of OCEQ in Lithuania, the questions were translated from Dutch to English and then from English to Lithuanian. To validate the Lithuanian version, the Lithuanian version was back-translated to English. We found no significant discrepancies between the two English versions. Two pediatric oncologists, a gynecologist, and two patients and their parents, all of whom were native Lithuanian speakers and could speak and understand English, reviewed the questionnaire. A native Dutch-speaking author compared the Dutch version with the Lithuanian version with the help of the English version. More information about the development of the questionnaire is provided in previous reports (22, 33). The study participants were provided with the Lithuanian version of the questionnaire (Supplementary Additional file 1). The English version is provided in Supplementary Additional file 2.

Fertility informing was defined as the delivery of information about fertility after cancer treatment by a dedicated oncofertility specialist (a medical doctor) to the family (one or both caregivers) of a child with cancer, irrespective of the potential infertility risk based on the anticipated treatment. Counseling was defined as additional referral to an expert fertility specialist to discuss the advantages and disadvantages of fertility preservation options. Counseling is offered primarily to children with a high risk of gonadal damage in countries with the necessary infrastructure. Caregivers evaluated education about the risk of gonadal damage and fertility-related questions as a part of oncofertility care before and after the implementation of routine fertility informing.

At the time of this study, fertility preservation was not available for girls at VUHSK; therefore, counseling was not offered to female patients at that time. For pubertal boys, semen preservation was available; however, they were referred directly to the fertility expert for counseling, thereby omitting routine informing; therefore, this subset of patients was not included in the present study.

### Ethics approval

This study was performed in line with the principles of the Declaration of Helsinki. The study was approved by Vilnius Regional Bioethics Committee (approval no 2020/10-1274-753).

### Gonadal damage risk stratification tool (triage)

In addition to the original gonadal damage risk stratification tool published by Máxima (31) and adapted for Lithuanian healthcare (33) (Supplementary Additional file 3), the risk of gonadal damage was evaluated according to the CED (32), the use of abdominal/testicular radiation, and ovarian surgery. According to IGHG recommendations, low gonadal damage risk (LGDR) for girls was defined as a CED of <4,000 mg/m^2^ and high gonadal damage risk (HGDR) as a CED of ≥6,000 mg/m^2^ (23). The LGDR for boys was defined as a CED of <4,000 mg/m^2^ (16) (Additional file 4). The risk of gonad damage was also considered high for boys and girls who underwent hematopoietic stem cell transplantation, total body irradiation, and radiotherapy to the gonads, in addition to ovariectomy and orchiectomy (16, 23).

### Study participants

#### Participants invited before the implementation of routine fertility informing

The baseline evaluation of the quality of oncofertility care before the implementation of standard fertility informing was conducted between 1 July 2021 and 1 July 2022. Parents of children treated for pediatric cancer (ICD-10-AM C00-96) were invited to participate in the study and complete the OCEQ. Baseline evaluation included parents of children aged <18 years who were diagnosed during this time frame and those diagnosed previously but continued treatment between 1 July 2021 and 1 July 2022. The OCEQ was completed during inpatient stays or outpatient visits during the study period, irrespective of the treatment phase. For accurate comparison, this baseline group included the cohort previously described by Stukaite-Ruibiene et al. (33).

All patients in the baseline group had not been specifically informed about potential fertility risk by a dedicated medical professional (33). As part of their standard of care, parents in the baseline group received a written informed consent form for cancer treatment in which all potential side effects of cancer treatment, including infertility, were listed. At that time, patients were not triaged to discuss fertility issues with a pediatric oncologist. This discussion, when it was conducted, was sporadic, not structured, and not documented in patients' medical history at that time. This group of patients was invited to evaluate their oncofertility care using the OCEQ. The respondents were also asked to self-estimate their fertility impairment risk. After completing the OCEQ, the study investigator triaged all participants to LGDR or HGDR retrospectively. The responses of the participants were compared with the correct infertility risk calculated by the study team using the gonadal risk stratification tool (33).

#### Routinely informed participants

Routine fertility information began on 1 July 2022. Until 1 July 2023, the program had been conducted sporadically; however, after 1 July 2023, fertility education was provided to most new patients by a single dedicated, trained team member (medical doctor). Newly diagnosed patients were identified. We aimed to prospectively triage all newly diagnosed patients using the gonadal damage risk stratification tool (Supplementary Additional file 4) and guide the parents regarding the anticipated risk of fertility impairment at the beginning of the children's treatment.

The families and patients with LGDR and those with HGDR were informed about fertility impairment. After a private conversation with the family, the dedicated team member provided supportive material (individualized leaflets; Supplementary Additional file 5) to all patients. Patients were encouraged to ask questions regarding fertility and preservation options. After 1 July 2023, we aimed to inform patients twice in cases of leukemia: first, at the beginning of the treatment to provide general information regarding fertility impairment without specifying gonadal damage risk because it could not be ascertained before the end of the induction therapy, and second, after the induction therapy when the exact treatment regimen was known to educate patients about individual gonadal damage risk. All patients and their families who had received the information were invited to participate in the study, and their caregivers were invited to complete the OCEQ. Patients who experienced disease recurrence and were re-informed about potential fertility damage as part of standard care were not included in this study.

### Data collection

After parents provided written informed consent to participate in the study and completed the questionnaire, patients' health data were collected from the electronic health records of our institution. To anonymize the data, each participant was assigned a code. Information collected included age at the time of diagnosis, the time from the date of diagnosis to the date of completing the questionnaire, the type of cancer, and its treatment. The anonymized information was documented in a Microsoft Excel database. The responses from the two respondent groups before and after the implementation of routine fertility education were compared and analyzed in January 2025.

### Statistical analyses

Normality of the distribution of continuous variables was evaluated using the Shapiro–Wilk test, which revealed that the data were not normally distributed. Therefore, to evaluate the characteristics of patients and responses to the questionnaires, we used descriptive statistics [medians, minimum and maximum ranges, and interquartile ranges (IQRs)] and the non-parametric Mann–Whitney *U*-test. To compare categorical variables, we used Pearson's chi-square test. The level of statistical significance was set at 5% (*p* < 0.05). SPSS Statistics version 17 (SPSS Inc., Chicago, IL, USA) was used for all quantitative analyses.

## Results

### Respondents invited before the implementation of routine fertility education

Between 1 July 2021 and 1 July 2022, 120 parents of patients were invited to participate in the study (Figure 1). Of the participants, 56 (46.7% parents of 28 boys and 28 girls) signed the informed consent form and completed the OCEQ. The other 64 invited parents or guardians (53.3%) refused to participate without specifying the reason. Therefore, their data could not be investigated. Six boys underwent semen cryopreservation during this period; however, their data were not analyzed in this study, as it focuses only on the quality of fertility information.

**Figure 1:**
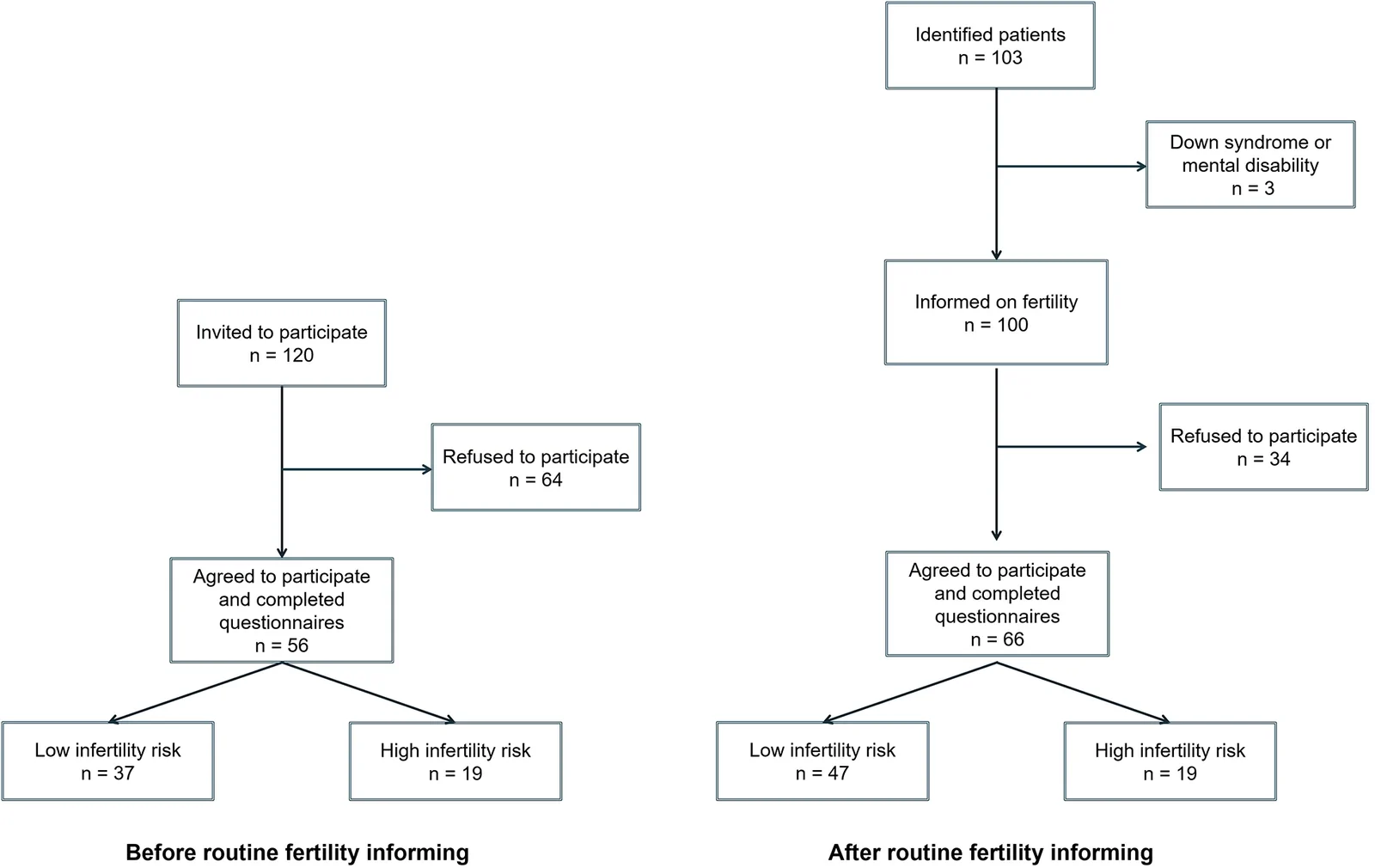
Flowchart of participant inclusion in the study.

### Respondents invited after the implementation of routine fertility informing

The second group consisted of patients who were identified as having a new cancer diagnosis after routine fertility informing was implemented (from 1 July 2022 to 1 September 2024). In total, 103 patients underwent treatment for childhood cancer. They had not previously been informed specifically about the risk of impaired fertility, and their parents had not completed the OCEQ (Figure 1). Of these patients, two were diagnosed with Down syndrome and one had severe cognitive disability; their parents were informed about fertility impairment during an initial conversation with the pediatric oncologist before cancer treatment when adverse effects of the treatment were described; however, their data were not included in this study. Other parents/caregivers and patients ≥12 years (the age at which children's informed consent to participate in clinical studies is considered legally valid in Lithuania) were provided information regarding fertility, and a dedicated physician emphasized their individual infertility risk. Seven families of children with acute lymphocytic leukemia (ALL) were informed of fertility risks twice: at the beginning of treatment and after the treatment regimen was established. In addition to parents of patients with new cancer diagnoses, all parents of other patients treated at the time of the study were informed about fertility risks. During routine fertility informing, none of the patients underwent fertility preservation.

Of the 100 informed parents of patients ≥12 years, 66 (66%; 35 boys and 31 girls) agreed to participate in the study, signed the informed consent form, and completed the OCEQ. Respondents recruited after the implementation of fertility informing agreed to participate in the study significantly more often than those recruited before the implementation (*p* < 0.01). Of the seven families who received fertility education twice, four agreed to participate and completed the OCEQ.

### Characteristics of respondents

Before the implementation of routine fertility informing, the median age of the patients at diagnosis was 6 years and ranged from 5 months to 17 years (IQR, 3–10 years; Table 1). After the implementation, the median age of the patients was 6 years and ranged from 1 month to 17.9 years (IQR, 2–11 years). Age at diagnosis in these two groups did not differ significantly (*p* = 0.82). The median ages at the completion of the questionnaire were 8 years (1–17.9 years; IQR, 6–12 years) before the implementation of the routine fertility informing and 6 years (4 months to 17.9 years; IQR, 3–12 years) after the implementation; differences between the median ages were not significant (*p* = 0.25). Before the routine informing, the median time from diagnosis to completion of the OCEQ was 19 months (22 days to 120 months; IQR, 8–30 months); after the implementation, the median time was 3 months (11 days to 80 months; IQR 1–11 months). The time from diagnosis to the completion of OCEQ was significantly shorter after the implementation (*p* < 0.001).

**Table 1:** Characteristics of the study participants before and after routine provision of fertility education.

Baseline characteristics	Risk of gonadal damage								
	Before routine fertility informing			After routine fertility informing			All		
	Low (*n* = 37)	High (*n* = 19)	Total (*n* = 56)	Low (*n* = 47)	High (*n* = 19)	Total (*n* = 66)	Low (*n* = 84)	High (*n* = 38)	Total (*n* = 122)
Age at diagnosis (years)									
Median	7	5	6	6	4	6	6	5	6
Range	1–17	0.4–16	0.4–17	0.08–17.9	1–17	0.08–17.9	0.08–17.9	0.4–17	0.08–17.9
IQR	4–10	3–10	3–10	2–11	2–13	2–11	3–10	2–12	2–11
Age at enrollment (years)									
Median	9	7	8	6	5	6	8	7	8
Range	1–17.8	1–17.9	1–17.9	0.3–17.9	1–17.9	0.3–17.9	0.3–17.9	1–17.9	0.3–17.9
IQR	7–12	5–11	6–12	4–12	2–14	3–12	5–12	3–13	4–12
Time from diagnosis to completion of OCEQ (months)									
Median	19	19	19	4	3	3	9	10	19
Range	0.07–120	0.06–73	0.06–120	0.13–80	0.03–60	0.03–80	0.07–120	0.03–73	0.07–120
IQR	12–30	4–29	8–30	1–11	1–18	1–11	2–20	3–27	1–11
Diagnosis, *n* (%)									
Hematologic malignancies									
Acute lymphoblastic leukemia	15 (40.5)	3 (15.8)	18 (32.1)	26 (55.3)	3 (15.8)	29 (43.9)	41 (48.8)	6 (15.8)	47 (38.5)
Acute myeloid leukemia	—	—	—	3 (6.4)	—	3 (4.5)	3 (3.6)	—	3 (2.5)
Other leukemias	1 (2.7)	—	1 (1.8)	2 (4.3)	2 (10.5)	4 (6.1)	3 (3.6)	2 (5.3)	5 (4.1)
Hodgkin lymphoma	1 (2.7)	3 (15.8)	4 (7.1)	5 (10.6)	1 (5.3)	6 (9.1)	6 (7.1)	4 (10.5)	10 (8.2)
Non-Hodgkin lymphoma	—	2 (10.5)	2 (3.6)	1 (2.1)	3 (15.8)	4 (6.1)	1 (1.2)	5 (13.2)	6 (4.9)
Langerhans cell histiocytosis	1 (2.7)	—	1 (1.8)	1 (2.1)	—	1 (1.5)	2 (2.4)	—	2 (1.6)
CNS tumors									
All CNS tumors	10 (27)	4 (21.1)	14 (25)	1 (2.1)	—	1 (1.5)	11 (13.1)	4 (10.5)	15 (12.3)
Solid tumors									
Neuroblastoma	2 (5.4)	1 (5.3)	3 (5.4)	1 (2.1)	5 (26.3)	6 (9.1)	3 (3.6)	6 (15.8)	9 (7.4)
Renal tumors	4 (10.8)	2 (10.5)	6 (10.7)	1 (2.1)	1 (5.3)	2 (3.0)	5 (6.0)	3 (7.9)	8 (6.6)
Osteosarcoma	1 (2.7)	—	1 (1.8)	4 (8.5)	—	4 (6.1)	5 (6.0)	—	5 (4.1)
Ewing sarcoma	—	1 (5.3)	1 (1.8)	—	—	—	—	1 (2.6)	1 (0.8)
Soft tissue sarcoma	1 (2.7)	2 (10.5)	3 (5.4)	—	3 (15.8)	3 (4.5)	1 (1.2)	5 (13.2)	6 (4.9)
Hepatoblastoma	1 (2.7)	—	1 (1.8)	—	—	—	1 (1.2)	—	1 (0.8)
Germ cell tumors	—	—	—	2 (4.3)	1 (5.3)	3 (4.5)	2 (2.4)	1 (2.6)	3 (2.5)
Retinoblastoma	—	1 (5.3)	1 (1.8)	—	—	—	—	1 (2.6)	1 (0.8)
Total	37	19	56	47	19	66	84	38	122

CNS, central nervous system; IQR, interquartile range; OCEQ, oncofertility care evaluation questionnaire.

Before the implementation, the number of boys and girls was equal (28 each); after the implementation, the patients consisted of 35 boys (53%) and 31 girls (47%; *p* = 0.74). Of the 63 boys in both groups, 40 (63.5%) were considered to have LGDR and 23 (36.5%) to have HGDR. Of the 59 girls in both groups, 45 (76.3%) were considered to have LGDR and 15 (25.4%) to have HGDR. The distribution of infertility risk did not differ between boys and girls (*p* = 0.19). Before the implementation, 37 (66.1%) of the 56 respondents (both boys and girls) were considered to have LGDR and 19 (33.9%) to have HGDR; after the implementation, 47 (71.2%) of the 66 respondents were considered to have LGDR and 19 (28.8%) to have HGDR (*p* = 0.54).

The most common diagnosis in both groups was ALL in 26 participants (55.3%) before the routine informing and in 41 patients (48.8%) after the implementation of routine informing. Before the implementation, most patients with HGDR were diagnosed with central nervous system (CNS) tumors (four of 19, 21.2%) and Hodgkin lymphoma (three of 19, 15.8%). After the implementation, most children with HGDR had neuroblastoma (five of 19, 26.3%; Table 1).

In the comparison of diagnoses between the two groups, before the implementation of routine infertility informing, 26 (46.4%) patients were diagnosed with hematologic malignancies and 30 (53.6%) with solid tumors, and after the implementation of routine infertility informing, 47 (71.2%) patients were diagnosed with hematologic malignancies and 19 (28.8%) with solid tumors (*p* < 0.01). Overall, in the hematologic malignancies group, 56 (76.7%) patients were classified as those at LGDR and 17 (23.3%) as those at HGDR, whereas in the solid tumors group, 28 (57.1%) were classified as those at LGDR and 21 (42.9%) as those at HGDR (*p* = 0.02) (Table 1).

### Responses to the oncofertility evaluation questionnaire

All responses to the OCEQ before and after the implementation of routine fertility informing are provided in Figure 2.

**Figure 2:**
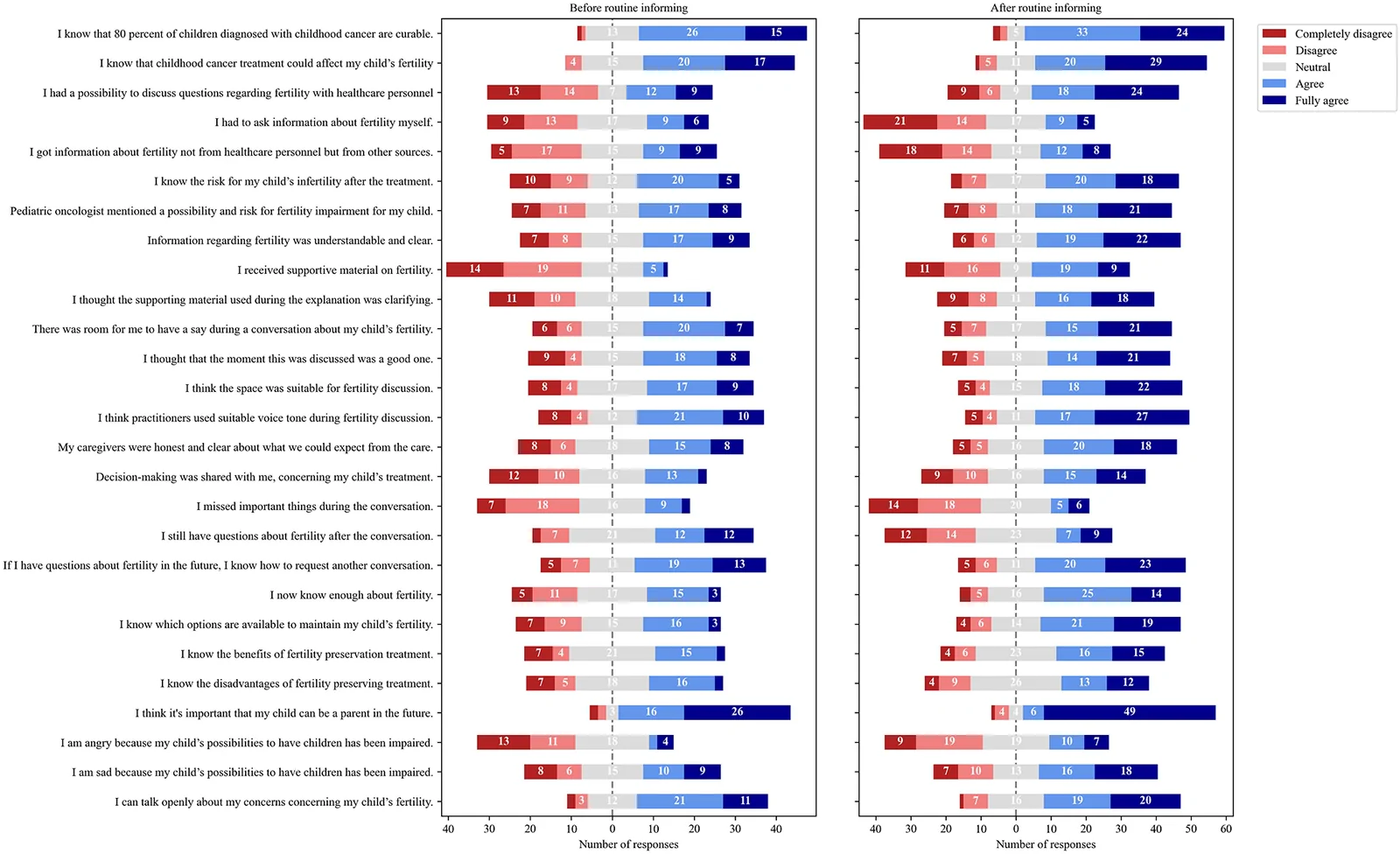
Responses to the oncofertility care evaluation questionnaire before and after implementation of routine fertility informing.

Before the implementation, 21 respondents (37.5%) agreed or fully agreed that they had an opportunity to discuss questions regarding fertility with healthcare personnel; after the implementation, 42 (63.6%) agreed that they had an opportunity to discuss questions regarding fertility with healthcare personnel (*p* = 0.002). Before the implementation, 20 (35.7%) agreed and 5 (8.9%) fully agreed that they were aware of the risk of infertility after the treatment. After the routine fertility education, 20 (30.3%) agreed and 18 (27.3%) fully agreed that they were aware of the risk (*p* = 0.007). Before the implementation, 25 (44.6%) respondents agreed that the pediatric oncologist mentioned the possibility of fertility impairment; after the implementation, 39 (59.1%) agreed that the pediatric oncologist mentioned the possibility of fertility impairment (*p* = 0.04). Before the implementation, 17 (30.4%) agreed and 9 (16.1%) fully agreed that the information regarding fertility was understandable and clear; after the implementation, 19 (28.8%) agreed and 22 (33.3%) fully agreed (*p* = 0.03). Regarding whether supportive material on fertility was provided, before the implementation, only 6 (10.7%) respondents stated that they received supportive material on fertility, whereas 28 (42.4%) respondents after the implementation stated that they received supportive material on fertility (*p* = 0.003). Before the implementation, 24 (42.9%) respondents still had questions about fertility after the conversation with the pediatric oncologist compared with 16 (24.2%) after the implementation (*p* = 0.003). More respondents thought they had adequate awareness of fertility after the implementation [39 (59.1%)] than before the implementation [18 (32.1%); *p* = 0.001]. Before the implementation, 18 (32.1%) respondents were aware of the options available to maintain their child's fertility compared with 40 (60.6%) after the implementation (*p* = 0.001; Figure 2).

Answers to the open-ended question “When is the best time to have a conversation about fertility?” were similar for the respondents surveyed before and after the implementation of routine fertility informing. Before the implementation, 23 (41.1%) respondents answered; of these, 20 (87%) stated that the best time for fertility counseling was before cancer treatment, whereas 3 (13%) stated that the information should be provided after treatment. After the implementation, 25 (37.9%) respondents answered this question; according to 19 (76%), the best time was before or early in treatment; 1 respondent (4%) stated that the conversation should be conducted during the patient's adolescence, 2 (8%) preferred after the end treatment, and 3 (12%) preferred in the middle of the treatment. Three respondents stated that fertility should be discussed repeatedly.

Before the implementation of routine fertility informing, 13 (23.2%) respondents stated that fertility was not discussed at all. After the routine informing implementation, despite the implementation and documentation of fertility education, 12 of 66 (18.2%) respondents forgot that it was provided.

### Self-evaluation of gonadal damage risk compared with evaluation by the stratification tool

The risks of gonadal damage according to the stratification tool and the respondents (self-evaluation) are compared in Table 2. More respondents correctly estimated their infertility risk after the routine fertility informing implementation than before the implementation.

**Table 2:** Comparison of the gonadal damage risk estimated by the study team and by respondents.

Respondents' estimated risk of gonadal damage	Risk of gonadal damage according to triage team^a^			
	Before implementation of routine informing (*n* = 56)		After implementation of routine informing (*n* = 66)	
	Low	High	Low	High
Low	5	3	30	—
High	3	5	2	12
Did not know	26	11	15	7
Did not answer	3	—	—	—

a Estimated by oncofertility care team.

Before the implementation, the majority of respondents answered that they did not know their children's infertility risk, which included 26 of 37 (70.3%) parents of patients with LGDR and 11 of 19 (57.9%) parents of patients with HGDR. The risk was estimated correctly by only 5 (13.5%) parents of patients with LGDR and 5 (26.3%) parents of patients with HGDR. Three (15.8%) parents of patients with HGDR thought that their children's infertility risk was low (Table 2).

After routine fertility informing, more than half (30 of 47, 63.8%) of parents of patients at LGDR correctly estimated their risk (compared with 13.5%). All four families diagnosed with ALL who were informed twice were attributed to LGDR, and all of them correctly identified their risk. Similarly, the majority (12 of 19, 63.2%) of the parents of patients at HGDR correctly identified their gonadal damage risk (compared with 26.3%). No parents of patients at HGDR thought that their risk was low. However, 15 of 47 (31.9%) parents of patients at LGDR (compared with 70.3%) and 7 of 19 (36.8%) parents of patients at HGDR (compared with 57.9%) still reported that they were not aware of the infertility risk after infertility education. Evaluation of LGDR significantly differed between the two groups; significantly more respondents with low risk correctly estimated their risk after the routine education (Bonferroni corrected *p* = 0.04).

## Discussion

Our results clearly demonstrated that following the implementation of routine fertility informing, more parents of patients were better informed about fertility and its impairment (Figure 2; Table 2). In addition, families' satisfaction with their children's care improved significantly, more parents were aware of their individual child's infertility risk, their questions about fertility were addressed, and the information had been provided carefully and appropriately (Figure 1). The evaluation of fertility information was based primarily on the information about gonadal toxicity that families received; however, other aspects of psychological importance, such as the time and place of fertility education and use of a suitable tone by the dedicated physician, were also included. Interestingly, after the implementation, significantly more respondents fully agreed that it was important that a child with cancer could have biological children in the future (Figure 1), indicating that fertility and personal risk information strengthened the significance of fertility education for patients and their parents. In addition, the percentage of parents of patients who agreed to participate in the study before and after the implementation differed significantly (46.7% vs. 66%). Families who received the information may have been more satisfied, and they tended to participate in the evaluation process more actively than previously. Notably, after the implementation, all parents of patients with HGDR were aware of their children's infertility risk (Table 2), indicating the success of the education.

In total, 103 patients with cancer were identified as eligible for this study, and the risk for infertility was calculated for all patients at our center from 1 July 2022 to 1 September 2024. Each year, approximately 50–60 new cases of childhood cancer are diagnosed and treated at VUHSK (22); thus, 103 is equivalent to two annual populations of new pediatric patients with cancer, and routine education about infertility would be useful for such a large number.

According to the IGHG guidelines, information about infertility risk should be provided as soon as possible after diagnosis by a dedicated pediatric oncologist, a fertility specialist, an endocrinologist, or a specialized nurse (16, 23). In our setting, families were educated adequately by a medical doctor with expertise in this topic. As demonstrated in the Netherlands, this information could also be provided by a dedicated nurse, nurse practitioner, or another healthcare professional together with, or in close connection with, the treating pediatric oncologist (31). This depends on the local availability of such professionals. For the next step, patients and their families should be counseled by a fertility expert who can guide the process of decision-making regarding fertility preservation.

The characteristics of the respondents before and after the implementation were comparable; thus, the results of the comparison are more reliable (Table 1). Ages at diagnosis and completion of questionnaires did not differ significantly. The numbers of boys and girls in both groups with LGDR and HGDR did not differ significantly. The most common diagnosis in both groups was ALL, which is also the most common childhood cancer (38). However, solid tumors, most of which were of the CNS, were diagnosed in significantly more patients before the implementation than after (Table 1). This difference can be attributed to the patients' invitation process. Most cases of CNS tumors were treated only by surgery in another department, and they may not have been identified in the inpatient setting; therefore, patients and their families may not have received fertility information in a timely manner. Because no informed consent forms were available in such cases, such data could not be analyzed. Before the implementation of routine fertility education, parents of patients in outpatient settings were also invited to participate in this study; therefore, more patients with CNS tumors were included. Both before and after the implementation, solid tumors, which are related primarily to higher CEDs, were diagnosed in most patients at HGDR (33) (Supplementary Additional file 4).

The time from the diagnosis to completion of questionnaires was significantly shorter for those who received routine fertility informing, which was a key difference between the two groups (Table 1). This finding may reflect the fact that after the implementation of routine informing, information was successfully provided early in the treatment, at a median of 3 months, as recommended by the guidelines (16, 23). This difference may be explained by the likelihood that respondents before the implementation forgot the information provided by a pediatric oncologist at the time of the treatment.

Of the informed respondents, 18.2% stated they did not have any conversation about fertility despite being informed. Parents of patients have been reported to recall only about 20% of the information provided during stressful situations (39, 40). Our previous study showed that families informed by only a pediatric oncologist were not provided enough supportive material (33); therefore, after the implementation, we distributed individualized leaflets containing general information about fertility and highlighted individual risk for infertility. After the implementation, significantly more parents agreed that supportive material was provided and that it was understandable and clear (Figure 1).

Although the perception of CCSs regarding fertility information is being investigated (26, 27, 30), routine fertility care is still not widely implemented (33, 41). To our knowledge, only one study, in the Netherlands, reported on a systematic investigation of the quality of routine oncofertility care in patients with childhood cancer; the Dutch version of our previously published questionnaire was used to evaluate the fertility counseling of children with Hodgkin lymphoma (41). In that study, 76% of respondents indicated that they had received clear information on fertility, compared with 62% in our study (Figure 2). In addition, 13% of the respondents in that study missed important information during the conversation, compared with 17% in our study. In both studies, 61% mentioned that fertility preservation had been discussed. In both the aforementioned study and our study, 24% of the respondents still had questions about fertility after the conversation. This finding indicates that one informative conversation may be insufficient. Our study population included patients with Hodgkin lymphoma and those with all childhood cancer types; however, we observed a similar trend in patient satisfaction regarding routine fertility informing.

As indicated by various guidelines, our findings showed that fertility preservation in patients with childhood cancer in real-life practice before treatment is important but challenging (16, 23, 42, 43). In addition, during this study, at our institution in Lithuania, girls had no opportunity for fertility preservation or referral for counseling by a fertility specialist. Ovarian tissue preservation was not feasible at that time, and the lack of this option may hamper the process of providing information. In addition, six pubertal boys underwent semen preservation before the implementation of routine fertility education; however, as mentioned earlier, they were not included in this study. Therefore, in the future, international guidelines as published by American Society for Reproductive Medicine (ASRM) stating that ovarian tissue cryopreservation should become available for all girls with cancer with a high fertility risk should be taken into account (44, 45). Early evidence on the efficacy and safety of these procedures from other countries should be shared and published to encourage surgeons and gynecologists to consider performing them in vulnerable children with cancer (31, 46–50).

However, the success of fertility informing also depends on organizational resources, availability of trained multidisciplinary teams, operating room access, and legal requirements. This is crucial because the topic of fertility preservation is relatively new, not just in Lithuania, where the Law for Assisted Reproduction was adopted in 2017 (51). Overall, it was only in 2019 that the ASRM stated clearly that ovarian tissue cryopreservation should also be offered as a standard of care for prepubertal and young adolescent girls (45). Since then, more countries have attempted, if ethically and legally allowed, to implement counseling and preservation as a standard of care for children and adults in oncology settings. Distinct regulatory frameworks on oncofertility practices in different countries are also important. Our study facilitated the development of standard operating procedures for fertility education of patients with childhood cancer at VUHSK, which had not been approved before the study. In some countries, such as France, informing patients about the risk of gonadal damage associated with cancer treatment, as well as discussing fertility preservation options, is a legal requirement. Other countries, such as Canada, have no national recommendations for fertility preservation. Regulatory differences may partly explain international variations in fertility counseling practices. French pediatric oncologists are reported to consider fertility preservation a moral duty and a standard of care, whereas the majority of Canadian pediatric oncologists are reported to consider it experimental and are more concerned about the risk of reintroducing malignant cells (52).

Because close collaboration between oncofertility specialists is crucial, a multidisciplinary team has been established at our institution, which is expected to result in more effective communication and timely fertility preservation as part of the oncofertility care plan, as pursued in other countries at present (31). The fact that parents of patients perceived fertility informing positively even without fertility preservation confirms the results of other studies and reinforces our activities to take these next steps (20, 21). Current reports of healthy newborns born from cryopreserved ovarian tissue harvested from prepubertal children are promising (53–63).

Patients were not re-triaged repeatedly in our study; however, research has shown that infertility risk can change later in treatment, particularly after relapses (31). We could not unfortunately build the infrastructure to systematically inform patients at relapse as of yet. Therefore, in the future, each patient treated at VUHSK will be re-educated if the anticipated fertility risk could change. The small sample size could be another limitation of this study; however, the number of participants included was equivalent to two annual populations of patients with pediatric cancer at our center.

To our knowledge, this is the first comparison of the perception of oncofertility care for patients with all types of childhood cancers before and after the implementation of routine fertility informing. The results clearly showed that routine provision of information improved parents' satisfaction with oncofertility care for patients with childhood cancer. Future efforts will include the establishment of timely referral of patients with HGDR to gynecologists and urologists for fertility preservation counseling. A five-step oncofertility care strategy for children with cancer has already been established (31). For patients and CCSs who underwent fertility preservation, researchers should also investigate whether their quality of life was improved not only by fertility informing but also by the fertility preservation procedure and the associated counseling.

## Conclusions

Routine gonadal damage risk triage and fertility informing for children with cancer was implemented successfully at VUHSK. Systematic information about fertility impairment resulting from childhood cancer treatment positively affected caregivers' satisfaction with oncofertility care, regardless of whether fertility preservation was possible. The families who received information about fertility and infertility risk from a dedicated physician were more satisfied with treatment than those who received only brief written information about fertility, among other side effects of chemotherapy. Establishment of an oncofertility team with specialists from various fields will enhance oncofertility care, including future fertility preservation.

## Data Availability

The raw data supporting the conclusions of this article will be made available by the authors, without undue reservation.
