# Supplementary material for: Parents’ perception of enhanced oncofertility care in childhood cancer patients following routine fertility informing
Source: Front Pediatr. 2026 Oct 21;14:1925381. doi: 10.3389/fped.2026.1925381 (PMC13731244; doi:10.3389/fped.2026.1925381)
Supplement: Supplementary file 1 [file Supplementaryfile1.docx]

**Supplemental Figure 1.** Informative individualized leaflet regarding patient gonadal damage risk.
