# Supplementary material for: Parents’ perception of enhanced oncofertility care in childhood cancer patients following routine fertility informing
Source: Front Pediatr. 2026 Oct 21;14:1925381. doi: 10.3389/fped.2026.1925381 (PMC13731244; doi:10.3389/fped.2026.1925381)
Supplement: Supplementary file 2 [file Table1.docx]

**Supplemental Table 1.** Lithuanian version of the oncofertility-care-evaluation questionnaire.

**Versija 3.0 2021-06-25**

Vilniaus universiteto ligoninėje Santaros klinikose vyksta Horizon 2020 „Mokslinių tyrimų ir švietimo bendradarbiavimo projektas, siekiant pagerinti vaikų, sergančių piktybiniais navikais, išgyvenamumą Lietuvoje (TREL) Nr.952438“. Projekto tikslas – pagerinti vaikų, sergančių onkologinėmis ligomis išgyvenamumą ir gyvenimo kokybę pasveikus.

Pasveikus nuo onkologinės ligos gali nukentėti vaisingumas – t .y. galimybė susilaukti vaikų. Vienas iš projekto tikslų – įvertinti, ar pacientų tėvai/globėjai ir patys pacientai tinkamai informuojami apie nevaisingumo riziką.

Prieš kurį laiką Jūsų vaikui buvo diagnozuota onkologinė liga. Prašytume pasidalinti savo patirtimi užpildant šį klausimyną, siekiant pagerinti konsultavimo dėl vaisingumo kokybę.

Aš esu: (pažymėkite	)

x

Vaiko mama	Vaiko tėtis

Auginu:

Mergaitę	Berniuką

Jūsų vaiko nevaisingumo rizika po onkologinės ligos gydymo:

Žema

Aukšta

Nežinau

Žemiau esantys teiginiai apibūdina konsultaciją dėl Jūsų vaiko vaisingumo. Pasirinkite vieną variantą prie kiekvieno teiginio. Nurodykite, kaip stipriai sutinkate su pateiktu teiginiu, apibraukdami skaičių prie atsakymo (1- visiškai nesutinku, 5 – visiškai sutinku).

**Bendri klausimai**

|  |  | Visiškai  nesutinku | Nesutinku | Nei sutinku, nei nesutinku | Sutinku | Visiškai  sutinku |
| --- | --- | --- | --- | --- | --- | --- |
| 1 | Aš žinau, kad nuo onkologinės ligos pasveiksta 80 proc. vaikų. | 1 | 2 | 3 | 4 | 5 |
| 2 | Aš žinau, kad onkologinės ligos gydymas gali pakenkti mano vaiko vaisingumui – t. y. galimybei susilaukti vaikų baigus gydymą. | 1 | 2 | 3 | 4 | 5 |
| 3 | Turėjau galimybę aptarti vaisingumo klausimus su medicinos personalu (gydytojais, slaugytojomis, psichologe). | 1 | 2 | 3 | 4 | 5 |

| 4 | Turėjau pats(-i) paprašyti suteikti informaciją apie vaisingumą. | 1 | 2 | 3 | 4 | 5 |
| --- | --- | --- | --- | --- | --- | --- |
| 5 | Informaciją apie vaisingumą gavau ne iš medikų (interneto, bendraujant su kitais tėvais ir pan.). | 1 | 2 | 3 | 4 | 5 |
| 6 | Aš žinau, kokia nevaisingumo rizika kilo mano vaikui dėl ligos gydymo. | 1 | 2 | 3 | 4 | 5 |

**Klausimai, susiję su pokalbiu su gydytoja onkohematologe**

|  |  | Visiškai  nesutinku | Nesutinku | Nei sutinku, nei nesutinku | Sutinku | Visiškai  sutinku |
| --- | --- | --- | --- | --- | --- | --- |
| 7 | Gydytoja onkohematologė minėjo galimą vaisingumo pažeidimą ir jo riziką mano vaikui | 1 | 2 | 3 | 4 | 5 |
| 8 | Pokalbių metu pateikta informacija apie vaisingumą buvo suprantama ir aiški. | 1 | 2 | 3 | 4 | 5 |
| 9 | Man buvo pateikta pagalbinė medžiaga apie vaisingumą (lankstinukai, knygos, nuorodos internete ar kt.) | 1 | 2 | 3 | 4 | 5 |
| 10 | Pateikta pagalbinė medžiaga buvo suprantama ir aiški. | 1 | 2 | 3 | 4 | 5 |
| 11 | Galėjau įsiterpti ir išsakyti savo nuomonę pokalbiųų dėl vaisingumo metu. | 1 | 2 | 3 | 4 | 5 |
| 12 | Manau, kad pokalbiai dėl vaisingumo įvyko  tinkamu metu. | 1 | 2 | 3 | 4 | 5 |
| 13 | Manau, kad pokalbiai dėl vaisingumo įvyko  tinkamoje aplinkoje. | 1 | 2 | 3 | 4 | 5 |
| 14 | Manau, kad gydytojai apie vaisingumą kalbėjo tinkamu tonu. | 1 | 2 | 3 | 4 | 5 |
| 15 | Gydytojai buvo atviri apie tai, ko galima tikėtis iš vaisingumo priežiūros. | 1 | 2 | 3 | 4 | 5 |
| 16 | Galėjau priimti sprendimą dėl savo vaiko vaisingumo išsaugojimo gydymo. | 1 | 2 | 3 | 4 | 5 |
| 17 | Man trūko svarbių dalykų per pokalbius dėl  vaisingumo. | 1 | 2 | 3 | 4 | 5 |
| 18 | Aš vis dar turiu klausimų apie vaisingumą. | 1 | 2 | 3 | 4 | 5 |
| 19 | Jeigu turėsiu klausimų apie vaisingumą ateityje, žinau, kur kreiptis. | 1 | 2 | 3 | 4 | 5 |

**Klausimai po pokalbio apie vaisingumą**

|  |  | Visiškai  nesutinku | Nesutinku | Nei sutinku, nei nesutinku | Sutinku | Visiškai  sutinku |
| --- | --- | --- | --- | --- | --- | --- |
| 20 | Aš dabar žinau pakankamai apie vaisingumą. | 1 | 2 | 3 | 4 | 5 |
| 21 | Aš žinau, kokios yra mano vaiko vaisingumo išsaugojimo galimybės. | 1 | 2 | 3 | 4 | 5 |
| 22 | Aš žinau apie vaisingumo išsaugojimo  gydymo privalumus. | 1 | 2 | 3 | 4 | 5 |
| 23 | Aš žinau apie vaisingumo išsaugojimo gydymo trūkumus. | 1 | 2 | 3 | 4 | 5 |
| 24 | Man svarbu, kad mano vaikas ateityje  galėtų būti mama/tėčiu. | 1 | 2 | 3 | 4 | 5 |
| 25 | Aš pykstu, nes sumažėjo mano vaiko galimybės turėti vaikų | 1 | 2 | 3 | 4 | 5 |
| 26 | Man liūdna, nes sumažėjo mano vaiko galimybės turėti vaikų | 1 | 2 | 3 | 4 | 5 |
| 27 | Aš galiu atvirai kalbėti apie susirūpinimą dėl  savo vaiko vaisingumo. | 1 | 2 | 3 | 4 | 5 |

## Ar yra dalykų, kurių jums trūko per pokalbius ar konsultacijas dėl vaisingumo?

Kaip	manote,	kada	yra	tinkamiausias	laikas	pokalbiui	apie	vaisingumą?

## Kaip manote, kas praėjo gerai per Jūsų vaiko vaisingumo priežiūrą?

Ar	turite	kitų	pastebėjimų	ar	patarimų	dėl	vaisingumo	priežiūros?

## Ačiū už skirtą laiką!
