# Supplementary material for: Parents’ perception of enhanced oncofertility care in childhood cancer patients following routine fertility informing
Source: Front Pediatr. 2026 Oct 21;14:1925381. doi: 10.3389/fped.2026.1925381 (PMC13731244; doi:10.3389/fped.2026.1925381)
Supplement: Supplementary file 3 [file Table2.docx]

**Supplemental Table 2.** English version of the oncofertility-care-evaluation questionnaire.

**Ver. 3.0 2021-06-25**

Vilnius University Hospital Santaros Klinikos is participating in Horizon 2020 project „Twinning in Research and Education to improve survival in Childhood Solid Tumours in Lithuania (TREL) Nr.952438“.The aim of the project – to improve outcomes and quality of life of children diagnosed with cancer.

After the cancer treatment your child‘s fertility could be impaired. One of the aims of the project – to evaluate and improve the quality of fertility counseling.We would like to use this questionnaire to find out how you experienced the counseling. I am: ( x )

Mother of child	Father of child

I raise:

Girl	Boy

Your child risk for infertility after childhood cancer treatment:

Low

High

I don‘t know

Statements below are related with fertility counseling. Please indicate to what extent you agree or disagree with these statements by circling one number next to the answer that applies to you (1 – completely disagree, 5 – strongly agree).

**General questions**

|  |  | Completely disagree | Disagree | Neutral | Agree | Strongly agree |
| --- | --- | --- | --- | --- | --- | --- |
| 1 | I know that 80 percent of children diagnosed with childhood cancer are curable. | 1 | 2 | 3 | 4 | 5 |
| 2 | I know that childhood cancer treatment could affect my child’s fertility (a possibility to have children after the treatment). | 1 | 2 | 3 | 4 | 5 |
| 3 | I had a possibility to discuss questions regarding fertility with healthcare personnel (doctors, nurses, psychologist). | 1 | 2 | 3 | 4 | 5 |
| 4 | I had to ask information about fertility myself. | 1 | 2 | 3 | 4 | 5 |
| 5 | I got information about fertility not from healthcare personnel but from other sources (Internet, other parents etc.) | 1 | 2 | 3 | 4 | 5 |
| 6 | I know the risk for my child’s infertility after the treatment. | 1 | 2 | 3 | 4 | 5 |

# This project has received funding from the European Union’s Horizon 2020 – Work Programme on Spreading Excellence and Widening Participation under grant agreement No 952438. ”

**Questions regarding conversation with pediatric oncologist:**

|  |  | Completely disagree | Disagree | Neutral | Agree | Strongly agree |
| --- | --- | --- | --- | --- | --- | --- |
| 7 | Pediatric oncologist mentioned a possibility and risk for fertility impairment for my child. | 1 | 2 | 3 | 4 | 5 |
| 8 | Information regarding fertility was understandable and clear. | 1 | 2 | 3 | 4 | 5 |
| 9 | I received supportive material on fertility (leaflets, books, website links etc.) | 1 | 2 | 3 | 4 | 5 |
| 10 | I thought the supporting material used during the explanation was clarifying. | 1 | 2 | 3 | 4 | 5 |
| 11 | There was room for me to have a say during a conversation about my child’s fertility. |  | 2 | 3 | 4 | 5 |
| 12 | I thought that the moment this was discussed was a good one. | 1 | 2 | 3 | 4 | 5 |
| 13 | I think the space was suitable for fertility discussion. | 1 | 2 | 3 | 4 | 5 |
| 14 | I think practitioners used suitable voice tone during fertility discussion. | 1 | 2 | 3 | 4 | 5 |
| 15 | My caregivers were honest and clear about what we could expect from the care. | 1 | 2 | 3 | 4 | 5 |
| 16 | Decision-making was shared with me, concerning my child’s treatment | 1 | 2 | 3 | 4 | 5 |
| 17 | I missed important things during the conversation. | 1 | 2 | 3 | 4 | 5 |
| 18 | I still have questions about fertility after the conversation. | 1 | 2 | 3 | 4 | 5 |
| 19 | If I have questions about fertility in the future, I know how to request another conversation. | 1 | 2 | 3 | 4 | 5 |

# This project has received funding from the European Union’s Horizon 2020 – Work Programme on Spreading Excellence and Widening Participation under grant agreement No 952438. ”

**Questions regarding conversation with pediatric oncologist:**

|  |  | Completely disagree | Disagree | Neutral | Agree | Strongly agree |
| --- | --- | --- | --- | --- | --- | --- |
| 20 | I now know enough about fertility. | 1 | 2 | 3 | 4 | 5 |
| 21 | I know which options are available to maintain my child’s fertility. | 1 | 2 | 3 | 4 | 5 |
| 22 | I know the benefits of fertility preservation treatment. | 1 | 2 | 3 | 4 | 5 |
| 23 | I know the disadvantages of fertility preserving treatment. | 1 | 2 | 3 | 4 | 5 |
| 24 | I think it's important that my child can be a parent in the future. | 1 | 2 | 3 | 4 | 5 |
| 25 | I am angry because my child’s possibilities to have children has been impaired | 1 | 2 | 3 | 4 | 5 |
| 26 | I am sad because my child’s possibilities to have children has been impaired. | 1 | 2 | 3 | 4 | 5 |
| 27 | I can talk openly about my concerns concerning my child’s fertility. | 1 | 2 | 3 | 4 | 5 |

Are there things you missed during the conversations regarding fertility or counseling?

What time do you think is the best time to have a conversation about fertility?

What do you think went well during fertility care?

Do you have any other additions or tips on fertility care?

Thank you for your time!
