# Supplementary material for: Parents’ perception of enhanced oncofertility care in childhood cancer patients following routine fertility informing
Source: Front Pediatr. 2026 Oct 21;14:1925381. doi: 10.3389/fped.2026.1925381 (PMC13731244; doi:10.3389/fped.2026.1925381)
Supplement: Supplementary file 4 [file Table3.docx]

**Supplemental Table 3.** Comparison of the original and amended gonadal damage risk stratification tools.

| **Original gonadal damage risk stratification tool** | | | | | **Amended gonadal damage risk stratification tool** | | | | | | **Differences from the original tool** |
| --- | --- | --- | --- | --- | --- | --- | --- | --- | --- | --- | --- |
| Tumor | Protocol | Treatment arm | CED mg/m^2^ | Female infertility risk | Tumor | Protocol | Treatment arm | CED mg/m^2^ | Female infertility risk by CED | Male infertility risk by CED | Males infertility risk by CED was added. |
| **Hematologic malignancies** | | | | | **Hematologic malignancies** | | | | | | - |
| Acute Lymphoblastic Leukemia | ALL-11 | SR, MR | 2000 | Low | Acute Lymphoblastic Leukemia | - | - | - | - | - | ALL-11 protocol was deleted as it is not used at Vilnius University Hospital Santaros Klinikos (VULSK). |
|  |  | HR 1-3 +SCT | 5600 + SCT | High |  | - | - | - | - | - |  |
|  |  | HR 1-6 + II | 9300 | High |  | - | - | - | - | - |  |
|  | Interfant06 | Germline LR/rearranged MR HR - SCT | 3000 | Low |  | Interfant06 | Germline LR/rearranged MR HR - SCT | 3000 | Low | Low | - |
|  |  | Rearranged MR HR + SCT | 3000+ SCT | High |  |  | Rearranged MR HR + SCT | 3000 + SCT | High | High |  |
|  | - | - | - | - |  | Interfant21 | MR-Low | 3000 | Low | Low | Interfant 21 protocol was added. |
|  | - | - | - | - |  |  | MR-High | 1000 | Low | Low |  |
|  | - | - | - | - |  |  | HR | SCT | High | High |  |
|  | EsPhALL | Arm A | 9000 | High |  | EsPhALL | Arm A | 9000 | High | High | - |
|  |  | Arm B | 3000 | Low |  |  | Arm B | 3000 | Low | Low |  |
|  |  | High risk arm | 3976 + SCT | High |  |  | High risk arm | 3976 + SCT | High | High |  |
|  |  | High risk arm | 5976 + SCT | High |  |  | High risk arm | 5976 + SCT | High | High |  |
|  | IntReALL | SR treatment arm A | 1976 | Low |  | - | - | - | - | - | InReALL protocol was deleted as it is not used at VULSK. |
|  |  | SR treatment arm A with SCT | 1976 + SCT | High |  | - | - | - | - | - |  |
|  |  | SR treatment arm B | 3400 | Low |  | - | - | - | - | - |  |
|  |  | SR treatment arm B with SCT | 3400 + SCT | High |  | - | - | - | - | - |  |
|  |  | HR | 1976 + SCT | High |  |  | - | - | - | - |  |
|  | ALLTogether | R1 standard, experimental | 0 | Low |  | ALLTogether | R1 standard, experimental | 0 | Low | Low | - |
|  |  | R2 standard, Exp arm A, Exp arm B | 3000 | Low |  |  | R2 standard, Exp arm A, Exp arm B | 3000 | Low | Low |  |
|  |  | R3 standard, Exp InO: IR-high risk | 2000 | Low |  |  | R3 standard, Exp InO: IR-high risk | 2000 | Low | Low |  |
|  |  | ABL HR allo-SCT (≥ 1-3 NOPHO blocks) | 2000 +SCT | High |  |  | ABL HR allo-SCT (≥ 1-3 NOPHO blocks) | 2000 + SCT | High | High |  |
|  |  | ABL IR-high | 2000 | Low |  |  | ABL IR-High | 2000 | Low | Low |  |
|  |  | HR BCP SCT 3 blocks | 4200 +SCT | High |  |  | HR BCP SCT 3 blocks | 4200 + SCT | High | High |  |
|  |  | HR BCP chemo 6 blocks | 7400 | High |  |  | HR BCP chemo 6 blocks | 7400 | High | High |  |
|  |  | HR T-cell without Nelarabine + HR blocks | 4200 | Intermediate |  |  | HR T-cell without Nelarabine + HR blocks | 4200 | Intermediate | High |  |
|  |  | HR T-cell with Nelarabine single | 1000 | Low |  |  | HR T-cell with Nelarabine single | 1000 | Low | Low |  |
|  |  | HR T-cell with Nelarabine single + HR blocks | 3200 | Low |  |  | HR T-cell with Nelarabine single + HR blocks | 3200 | Low | Low |  |
|  |  | HR T-cell with Nelarabine addition | 2000 | Low |  |  | HR T-cell with Nelarabine addition | 2000 | Low | Low |  |
|  |  | HR T-cell with Nelarabine addition + HR blocks | 4200 | Intermediate |  |  | HR T-cell with Nelarabine addition + HR blocks | 4200 | Intermediate | High |  |
|  |  | DS-SR | 1000 | Low |  |  | DS-SR | 1000 | Low | Low |  |
|  |  | DS-IR, DS-HR | 3000 | Low |  |  | DS-IR, DS-HR | 3000 | Low | Low |  |
| LCH (Langerhans Cell Histiocytosis) | LCH IV | Stratum 1 group 1 (MS-LCH) arm A / B / C / D | 0 | Low | LCH (Langerhans Cell Histiocytosis) | LCH IV | Stratum 1 group 1 (MS-LCH) arm A / B / C / D | 0 | Low | Low | - |
|  |  | Stratum 1 group 2 (SS-LCH) | 0 | Low |  |  | Stratum 1 group 2 (SS-LCH) | 0 | Low | Low |  |
|  |  | Stratum 2 | 0 | Low |  |  | Stratum 2 | 0 | Low | Low |  |
|  |  | Stratum V without clinical neurodegeneration | 0 | Low |  |  | Stratum V without clinical neurodegeneration | 0 | Low | Low |  |
|  |  | Stratum V with clinical neurodegeneration | 0 | Low |  |  | Stratum V with clinical neurodegeneration | 0 | Low | Low |  |
| Hodgkin lymphoma | EuroNet-PHL-C2 | TL1 | 1000 | Low | Hodgkin lymphoma | EuroNet-PHL-C2 | TL1 | 1000 | Low | Low | - |
|  |  | TL2 | 2000 | Low |  |  | TL2 | 2000 | Low | Low |  |
|  |  | TL2 intensified | 2500 | Low |  |  | TL2 intensified | 2500 | Low | Low |  |
|  |  | TL3 | 4000 | Intermediate |  |  | TL3 | 4000 | Intermediate | High |  |
|  |  | TL3 intensified | 5000 | Intermediate |  |  | TL3 intensified | 5000 | Intermediate | High |  |
| Non-B NHL (Non-Hodgkin Lymphoma) | Euro LB-02 | T-Cell LL stage I-II | 2000 | Low | Non-B NHL (Non-Hodgkin Lymphoma) | Euro LB-02 | T-Cell LL stage I-II | 2000 | Low | Low | - |
|  |  | T-Cell LL stage III-IV | 3000 | Low |  |  | T-Cell LL stage III-IV | 3000 | Low | Low |  |
|  |  | Non-T-Cell LL stage I-II | 2000 | Low |  |  | Non-T-Cell LL stage I-II | 2000 | Low | Low |  |
|  |  | Non-T-Cell LL stage III-IV | 3000 | Low |  |  | Non-T-Cell LL stage III-IV | 3000 | Low | Low |  |
| - | - | - | - | - | LBL (Lymphoblastic lymphoma) | EICNHL | Standard risk group, I ir II stages pB-LBL | 2000 | Low | Low | EICNHL protocol for LBL (Lymphoblastic lymphoma) was added. |
| - | - | - | - | - |  |  | High risk group, III and IV stages pB-LBL ir T-LBL (all stages) | 3000 | Low | Low |  |
| B-NHL/B-ALL (B-cell Non-Hodgkin Lymphoma/  acute lymphoblastic leukemia) | SKION B-NHL/B-ALL 2008 | Group A | 3000 | Low | B-NHL/B-ALL  (B-cell Non-Hodgkin Lymphoma/  acute lymphoblastic leukemia) | - | - | - | - | - | SKION B-NHL/B-ALL 2008 protocol was deleted as it is not used at VULSK. |
|  |  | Group B | 3300 | Low |  | - | - | - | - | - |  |
|  |  | Group C1 | 6800 | High |  | - | - | - | - | - |  |
|  |  | Group C2 | 6800 | High |  | - | - | - | - | - |  |
|  | - | - | - | - |  | NHL-BFM | R1 | 1976 | Low | Low | NHL-BFM protocol was added. |
|  | - | - | - | - |  |  | R2 | 4352 | Intermediate | High |  |
|  | - | - | - | - |  |  | R3-R4-CNS- | 4352 | Intermediate | High |  |
|  | - | - | - | - |  |  | R4-CNS+ | 4352 | Intermediate | High |  |
|  | - | - | - | - |  | EICNHL | A | 3000 | Low | Low | EICNHL protocol for B-NHL/B-ALL was added. |
|  | - | - | - | - |  |  | B I | 3300 | Low | Low |  |
|  | - | - | - | - |  |  | B II (High risk) | 3300 | Low | Low |  |
|  | - | - | - | - |  |  | C1 | 5800 | Intermediate | High |  |
|  | - | - | - | - |  |  | C3 | 5800 | Intermediate | High |  |
|  | Inter-B-NHL ritux | Group B HR | 3300 | Low |  | Inter-B-NHL ritux | Group B HR | 3300 | Low | Low | - |
|  |  | Group C1 | 5800 | Intermediate |  |  | Group C1 | 5800 | Intermediate | High |  |
|  |  | Group C3 | 5800 | Intermediate |  |  | Group C3 | 5800 | Intermediate | High |  |
|  |  | PMLBL | 4500 | Intermediate |  |  | PMLBL | 4500 | Intermediate | High |  |
| Anaplastic Large Cell Lymphoma (ALCL) | ALCL | LR | 3352 | Low | Anaplastic Large Cell Lymphoma (ALCL) | ALCL | LR | 3352 | Low | Low | - |
|  |  | SR arm 1 SR arm 3, HR arm 1, HR arm 2, HR arm 3, HR arm 4 | 6328 | High |  |  | SR arm 1 SR arm 3, HR arm 1, HR arm 2, HR arm 3, HR arm 4 | 6328 | High | High |  |
|  | - | - | - | - |  | EICNHL | Isolated skin ALCL | 0 | Low | Low | EICNHL protocol for Anaplastic Large Cell Lymphoma was added. |
|  | - | - | - | - |  |  | Systemic ALCL: completely resected | 3352 | Low | High |  |
|  | - | - | - | - |  |  | Systemic ALCL: all other stages | 6328 | High | High |  |
|  | - | - | - | - |  |  | Patients with CNS+ | 4352 | Intermediate | Low |  |
| Acute Myeloid Leukemia | Nopho DBH AML 2012 | SR | 0 | Low | Acute Myelod Leukemia | Nopho DBH AML  2012 | SR | 0 | Low | Low | - |
|  |  | HR | 0+SCT | High |  |  | HR | 0+SCT | High | High |  |
| Acute Promyelocytic  Leukemia | ICC APL 01 | SR MRD- / SR MRD+ / HR | 0 | Low | Acute Promyelocytic Leukemia | ICC APL 01 | SR MRD- / SR MRD+ / HR | 0 | Low | Low | - |
|  | ICC APL 02 | SR, HR | 0 | Low |  | ICC APL 02 | SR, HR | 0 | Low | Low |  |
| **Solid tumors** | | | | | **Solid tumors** | | | | | | - |
| Neuroblastoma | DCOG NBL 2009 | OG without N4 | 0 | Low | Neuroblastoma | - | - | - | - | - | DCOG NBL 2009 and DCOG NBL 2009 <1yr protocols were deleted as it is not used at VULSK. |
|  |  | OG with 1x N4 | 2100 | Low |  | - | - | - | - | - |  |
|  |  | OG with 2x N4 | 4200 | Intermediate |  | - | - | - | - | - |  |
|  |  | OG with 3x N4 | 6300 | High |  | - | - | - | - | - |  |
|  |  | OG with 4x N4 | 8400 | High |  | - | - | - | - | - |  |
|  |  | MR without N4 | 10290 | High |  | - | - | - | - | - |  |
|  |  | MR with N4 | 18690 | High |  | - | - | - | - | - |  |
|  |  | HR without N4 | 12690 | High |  | - | - | - | - | - |  |
|  |  | HR with N4 | 21090 | High |  | - | - | - | - | - |  |
|  | DCOG NBL 2009 <1yr | OG with 1x N4 <1yr | /kg | Low |  | - | - | - | - | - |  |
|  |  | OG with 2x N4 <1yr, 3x N4 <1yr, 4x N4 <1yr | /kg | Intermediate |  | - | - | - | - | - |  |
|  |  | MR without N4 <1yr, with N4 <1yr | /kg | High |  | - | - | - | - | - |  |
|  |  | HR without N4 <1yr, with N4 <1yr | /kg | High |  | - | - | - | - | - |  |
|  | - | - | - | - |  | SIOPEN European Low and Intermediate Risk Neuroblastoma (LINES) | LR group 1 | 0 | Low | Low | SIOPEN European Low and Intermediate Risk Neuroblastoma (LINES) protocol was added. |
|  | - | - | - | - |  |  | LR group 2 2xVP/CARBO | 0 | Low | Low |  |
|  | - | - | - | - |  |  | LR group 2 2xVP/CARBO +2x CADO | 3000 | Low | Low |  |
|  | - | - | - | - |  |  | LR group 3 4x VP/CARBO | 0 | Low | Low |  |
|  | - | - | - | - |  |  | LR group 3 2x VP/CARBO + 2x CADO | 3000 | Low | Low |  |
|  | - | - | - | - |  |  | LR group 4 | 0 | Low | Low |  |
|  | - | - | - | - |  |  | LR group 5 2xVP/CARBO | 0 | Low | Low |  |
|  | - | - | - | - |  |  | LR group 5 2xVP/CARBO +2x CADO | 3000 | Low | Low |  |
|  | - | - | - | - |  |  | LR group 6 4x VP/CARBO | 0 | Low | Low |  |
|  | - | - | - | - |  |  | LR group 6 2x VP/CARBO + 2x CADO | 3000 | Low | Low |  |
|  | - | - | - | - |  |  | IR group 7 4x VP/CARBO | 0 | Low | Low |  |
|  | - | - | - | - |  |  | IR group 7 2x VP/CARBO + 2x CADO | 3000 | Low | Low |  |
|  | - | - | - | - |  |  | IR group 8 2x VP/CARBO + 2x CADO + 1xVP/CARBO + 1xCADO | 4500 | Intermediate | High |  |
|  | - | - | - | - |  |  | IR group 8 2x VP/CARBO + 2x CADO + 2xCADO | 6000 | High | High |  |
|  | - | - | - | - |  |  | IR group 9 2x VP/CARBO + 2x CADO + 1xVP/CARBO + 1xCADO | 4500 | Intermediate | High |  |
|  | - | - | - | - |  |  | IR group 10 4x VP/CARBO | 0 | Low | Low |  |
|  | - | - | - | - |  |  | IR group 10 2x VP/CARBO + 2xCADO | 3000 | Low | Low |  |
|  | - | - | - | - |  |  | IR group 10 2x VP/CARBO + 4x CADO | 6000 | High | High |  |
|  | - | - | - | - |  |  | IR group 10 4x VP/CARBO + 4x CADO | 6000 | High | High |  |
|  | - | - | - | - |  | HR-NBL-1.8/SIOPEN | BuMel any weight | >12000 | High | High | HR-NBL-1.8/SIOPEN protocol was added. |
| Ewing | Ewing 2008 | R1 female | 25176 | High | Ewing | Ewing 2008 | R1 female | 25176 | High | High | - |
|  |  | R3 | 25176 | High |  |  | R3 | 25176 | High | High |  |
|  |  | R3 + TreoMel | 30776 | High |  |  | R3 + TreoMel or BuMel | 30776 | High | High |  |
|  | - | - | - | - |  | Ewing 2012 | Arm A R2 VAC | 25140 | High | High | Ewing 2012 protocol was added. |
|  | - | - | - | - |  |  | BuMel any weight | >23000 | High | High |  |
|  | - | - | - | - |  |  | Arm A R2 VAI | 24888 | High | High |  |
|  | - | - | - | - |  |  | Arm B R2 IEVC | 23772 | High | High |  |
|  | - | - | - | - |  |  | Arm B R2 BuMel any weight | >23000 | High | High |  |
| Osteosarcoma | EURAMOS 1 | MAP | 0 | Low | Osteosarcoma | EURAMOS 1 | MAP | 0 | Low | Low | - |
|  |  | MAPIE | 14640 | High |  |  | MAPIE | 14640 | High | High |  |
| Renal tumors | UMBRELLA 2016/SIOP 2001** | AV + AVD, AV + AV1, AV + AV2 | 0 | Low | Renal tumours | UMBRELLA 2016/SIOP 2001 | AV + AVD, AV + AV1, AV + AV2 | 0 | Low | Low | - |
|  |  | AV + HR | 8100 | High |  |  | AV + HR | 8100 | High | High |  |
| Rhabdoid tumors of the kidney (RTK) or of soft tissue (MRT) | EpSSG NRSTS 2005 | Cyclophosphamide | 17000 | High | Rhabdoid tumors of the kidney (RTK) or of soft tissue (MRT) | EpSSG NRSTS 2005 | Cyclophosphamide | 17000 | High | High | - |
|  | EURHAB <18 mo | 3x DOX, 3x ICE, 3xVCA | 8892 | High |  | EURHAB <18 mo | 3x DOX, 3x ICE, 3xVCA | 8892 | High | High |  |
|  | EURHAB <18 mo HD | 2x DOX, 2x ICE, 2x VCA + CARBO Thiotepa | 50928 | High |  | EURHAB <18 mo HD | 2x DOX, 2x ICE, 2x VCA + CARBO Thiotepa | 50928 | High | High |  |
|  | EURHAB >18 mo | 3x DOX, 3x ICE, 3xVCA + RT | 8892 | High |  | EURHAB >18 mo | 3x DOX, 3x ICE, 3xVCA + RT | 8892 | High | High |  |
|  | EURHAB >18 mo HD | 2x DOX, 2x ICE, 2x VCA + CARBO Thiotepa + RT | 50928 | High |  | EURHAB >18 mo HD | 2x DOX, 2x ICE, 2x VCA + CARBO Thiotepa + RT | 50928 | High | High |  |
| NRSTS | EpSSG NRSTS 2005 | 3x ifosfamide | 6588 | High | NRSTS* | EpSSG NRSTS 2005 | 3x ifosfamide | 6588 | High | High | - |
| (Non-Rhabdomyosarcoma Soft |  | 4x ifosfamide | 8784 | High | (Non-Rhabdomyosarcoma Soft |  | 4x ifosfamide | 8784 | High | High |  |
| Tissue Sarcoma) |  | 5x ifosfamide | 10980 | High | Tissue Sarcoma) |  | 5x ifosfamide | 10980 | High | High |  |
|  |  | 6x ifosfamide | 13176 | High |  |  | 6x ifosfamide | 13176 | High | High |  |
| Soft tissue sarcomas | EpSSG RMS2005 | LR subgroup A | 0 | Low | Soft tissue sarcomas | EpSSG RMS 2005 | LR subgroup A | 0 | Low | Low | - |
|  |  | SR subgroup B | 5800 | Intermediate |  |  | SR subgroup B | 5800 | Intermediate | High |  |
|  |  | SR subgroup C (9x Ifosfamide) | 13176 | High |  |  | SR subgroup C (9x Ifosfamide) | 13176 | High | High |  |
|  |  | SR subgroup C (5xIfosfamide) | 7320 | High |  |  | SR subgroup C (5xIfosfamide) | 7320 | High | High |  |
|  |  | SR subgroup D (9x Ifosfamide) | 13176 | high |  |  | SR subgroup D (9x Ifosfamide) | 13176 | High | High |  |
|  |  | HR and group A + group C | 13176 | high |  |  | HR ir group A + group C | 13176 | High | High |  |
|  |  | HR and group A + group D | 17376 | High |  |  | HR ir group A + group D | 17376 | High | High |  |
|  |  | HR and group B + group C | 13176 | High |  |  | HR ir group B + group C | 13176 | High | High |  |
|  |  | HR and group B + group D, VHR | 17376 | High |  |  | HR ir group B + group D, VHR | 17376 | High | High |  |
|  | - | - | - | - |  | CWS-guidance-2014 | RMS Low risk subgroup A | 0 | Low | Low | CWS-guidance-2014 protocol was added. |
|  | - | - | - | - |  |  | RMS standard risk subgroup B | 5856 | Intermediate | High |  |
|  | - | - | - | - |  |  | RMS standard risk subgroup C without RT | 13176 | High | High |  |
|  | - | - | - | - |  |  | RMS standard risk subgroup C with RT | 7320 | High | High |  |
|  | - | - | - | - |  |  | RMS standard risk subgroup D | 13176 | High | High |  |
|  | - | - | - | - |  |  | RMS High risk subgroups E, F, G | 13176 | High | High |  |
|  | - | - | - | - |  |  | RMS very High risk subgroup H | 13176 | High | High |  |
|  | - | - | - | - |  |  | “RMS-like” (SySa, STET (EES, pNET), UDS) | 13176 | High | High |  |
|  | - | - | - | - |  |  | “Non-RMS-like” (NRTS) Low risk | 0 | Low | Low |  |
|  | - | - | - | - |  |  | “Non-RMS-like” (NRTS) Intermediate risk | 0 | Low | Low |  |
|  | - | - | - | - |  |  | “Non-RMS-like” (NRTS) High risk | 13176 | High | High |  |
|  | - | - | - | - |  |  | Metastatic soft tissue tumours | 13176 | High | High |  |
|  | - | - | - | - |  |  | CWS-proposal for angiosarcoma | 6000 | High | High |  |
| Germ cell tumors | SIOP CNS GCT II | - | - | - | Germ cell tumors | SIOP CNS GCT II | CCLG | 4392 | Intermediate | High | CCLG and 2xPEI+2xHD-PEI (High risk non-germinoma) treatment arms of SIOP CNS GCT II protocol was added. |
|  |  | NGGCT | 7320 | High |  |  | NGGCT | 7320 | High | High |  |
|  |  | - | - | - |  |  | 2xPEI+2x HD-PEI (High risk non-germinoma) | 8540 | High | High |  |
|  | - | - | - | - |  | PEB+JEB blocks | Low risk | 0 | Low | Low | PEB + JEB blocks for germ cells tumors were added. |
|  | - | - | - | - |  |  | Intermediate risk | 0 (cis/carboplatin) | Low | Low |  |
|  | - | - | - | - |  |  | High risk | 0 (cis/carboplatin) | Low | Low |  |
| Liver tumors | PHITT | Group A1 very low risk HB | 0 | Low | Liver tumors | PHITT | Group A1 very Low risk HB | 0 | Low | Low | - |
| Hepatocellular carcinoma |  | Group A2 very low risk HB | 0 (cisplatin) | Low | Hepatocellular carcinoma |  | Group A2 very Low risk HB | 0 (cisplatin) | Low | Low |  |
|  |  | Group B1 Low risk HB / B2 | 0 (cisplatin) | Low |  |  | Group B1 Low risk HB / B2 | 0 (cisplatin) | Low | Low |  |
|  |  | Group C intermediate risk SIOPEL3HR / C5VD/ CDDP-M | 0 (cisplatin) | Low |  |  | Group C Intermediate risk SIOPEL3HR / C5VD/ CDDP-M | 0 (cisplatin) | Low | Low |  |
|  |  | Group D1 high risk HB SIOPEL4, D2 high risk HB CDCE, CDVI | 0 (cis/carboplatin) | Low |  |  | Group D1 High risk HB SIOPEL4, D2 High risk HB CDCE, CDVI | 0 (cis/carboplatin) | Low | Low |  |
|  |  | Group E1 resected HCC | 0 | Low |  |  | Group E1 resected HCC | 0 | Low | Low |  |
|  |  | Group E2 resected HCC PLADO | 0 (cisplatin) | Low |  |  | Group E2 resected HCC PLADO | 0 (cisplatin) | Low | Low |  |
|  |  | Group F unresected/metastatic PLADO sorafenib, GEMOX | 0 (cisplatin) | Low |  |  | Group F not resected/metastatic PLADO sorafenib, GEMOX | 0 (cisplatin) | Low | Low |  |
| **Brain tumors** | | | | | **Brain tumours** | | | | | | - |
| Opticus glioma | SIOP LGG 2004 | Vincristine, carboplatin, etoposide. (In case of allergy: cyclo) | 0 | Low | Opticus glioma | SIOP LGG 2004 | Vincristine, carboplatin, etoposide (if allergy: cyclo) | 0 | Low | Low | - |
| Intradural-extramedulary tumor | HIT-MED + SCT |  | 49500 | High | Intradural-extramedulary tumor | HIT-MED + SCT |  | 49500 | High | High | - |
| Medulloblastoma | SR ACNS0331 | Cyclophosphamide, lomustine | 13200 | High | Medulloblastoma | SR ACNS0331 | Cyclophosphamide, lomustine | 13200 | High | High | - |
|  | HR ACNS0332 | cyclophosphamide | 12000 | High |  | HR ACNS0332 | cyclophosphamide | 12000 | High | High | - |
|  | PNET 5 | MB-SR / MB-WNT-HR(>16years) | 17600 | High |  | PNET 5 | MB-SR / MB-WNT-HR(>16years) | 17600 | High | High | - |
|  |  | MB-WNT-HR (<16years) | 13200 | High |  |  | MB-WNT-HR (<16years) | 13200 | High | High |  |
|  |  | MB-SHH-TP53: No alkylating agents | 0 | Low |  |  | MB-SHH-TP53: no alkylating agents | 0 | Low | Low |  |
|  | - | - | - | - |  | HIT-MED Guidance | 3x SKK | 7200 | High | High | HIT-MED Guidance protocol was added. |
|  | - | - | - | - |  |  | 3x SKK+ 2x mSKK | 12000 | High | High |  |
|  | - | - | - | - |  |  | 3x intensified induction + 1^st^ HDCT + 2^nd^ HDCT + 6x maintenance | 4500+intensified induction | High | High |  |
|  | - | - | - | - |  |  | RT + 8x maintenance | 0 (cisplatin) | Low | Low |  |
|  | - | - | - | - |  |  | 2x SKK + RT + 4x maintenance | 4800 | Intermediate | High |  |
| AT/RT (Atypical  teratoid/rhabdoid tumors) | EURHAB <18 mo | 3x DOX, 3x ICE, 3xVCA | 8892 | High | AT/RT (Atypical teratoid/rhabdoid tumors) | EURHAB <18 mo | 3x DOX, 3x ICE, 3xVCA | 8892 | High | High | - |
|  | EURHAB <18 mo HD | 2x DOX, 2x ICE, 2x VCA + CARBO Thiotepa | 50928 | High |  | EURHAB <18 mo HD | 2x DOX, 2x ICE, 2x VCA + CARBO Thiotepa | 50928 | High | High |  |
|  | EURHAB >18 mo | 3x DOX, 3x ICE, 3xVCA + RT | 8892 | High |  | EURHAB >18 mo | 3x DOX, 3x ICE, 3xVCA + RT | 8892 | High | High |  |
|  | EURHAB >18 mo HD | 2x DOX, 2x ICE, 2x VCA + CARBO Thiotepa + RT | 50928 | High |  | EURHAB >18 mo HD | 2x DOX, 2x ICE, 2x VCA + CARBO Thiotepa + RT | 50928 | High | High |  |
| Dysgerminoma | WHO IV SIOP CNS GCT II HR-non-germinoma | PEI | 8540 | High | - | - | - | - | - | - | WHO IV SIOP CNS GCT II HR-non-germinoma protocol was deleted as it is not used at VULSK. |
| - | - | - | - | - | Ependymoma | HIT-MED Guidance | 3x SKK+2x mSKK | 12000 | High | High | HIT-MED Guidance protocol was ependymoma was added. |
| - | - | - | - | - |  |  | Local RT | 0 | Low | Low |  |
| - | - | - | - | - |  |  | 2x mSKK + RT | 4800 | Intermediate | High |  |
| - | - | - | - | - | Pineoblastoma | HIT-MED Guidance | CARBO/ETO-96h induction + 1st HDCT + 2nd HDCT + 6x maintenance) | 4500 | Intermediate | High | HIT-MED Guidance protocol for pineoblastoma was added. |
| - | - | - | - | - |  |  | RT + 8x maintenance | 0 (cisplatin) | Low | Low |  |
| - | - | - | - | - |  |  | 2x SKK + RT + 4x maintenance | 4800 | Intermediate | High |  |
| High grade glioma, Pons glioma | ACNS0126 | Temozolamide | 0 | Unknown | High grade glioma, pons glioma | ACNS0126 | Temosolamide | 0 | Unknown | Unknown | - |
| HGG (High grade glioma) | Infant HGG 2013/HIT SKK | Elements IIs IIIs/1 IIIs/2 IVs | 7200 | High | HGG (High grade glioma) | Infant HGG 2013/HIT SKK | Elements IIs IIIs/1 IIIs/2 IVs | 7200 | High | High | - |
