# Supplementary material for: Parents’ perception of enhanced oncofertility care in childhood cancer patients following routine fertility informing
Source: Front Pediatr. 2026 Oct 21;14:1925381. doi: 10.3389/fped.2026.1925381 (PMC13731244; doi:10.3389/fped.2026.1925381)
Supplement: Supplementary file 5 [file Table4.docx]

**Supplemental Table 4. Gonadal damage risk stratification tool (triage)^*^.**

| Tumor | Protocol | Treatment arm | CED mg/m^2^** | Female infertility risk by CED*** | Male infertility risk by CED**** |
| --- | --- | --- | --- | --- | --- |
| ematologic malignancies | | | | | |
| Acute lymphoblastic | Interfant06 | Germline LR/rearranged MR HR - SCT | 3000 | Low | Low |
| leukemia |  | Rearranged MR HR + SCT | 3000 + SCT | High | High |
|  | Interfant21 | MR-Low | 3000 | Low | Low |
|  |  | MR-High | 1000 | Low | Low |
|  |  | HR | SCT | High | High |
|  | EsPhALL | Arm A | 9000 | High | High |
|  |  | Arm B | 3000 | Low | Low |
|  |  | High risk arm | 3976 + SCT | High | High |
|  |  | High risk arm | 5976 + SCT | High | High |
|  | ALLTogether | R1 standard, experimental | 0 | Low | Low |
|  |  | R2 standard, Exp arm A, Exp arm B | 3000 | Low | Low |
|  |  | R3 standard, Exp InO: IR-high risk | 2000 | Low | Low |
|  |  | ABL HR allo-SCT (≥ 1-3 NOPHO blocks) | 2000 + SCT | High | High |
|  |  | ABL IR-High | 2000 | Low | Low |
|  |  | HR BCP SCT 3 blocks | 4200 + SCT | High | High |
|  |  | HR BCP chemo 6 blocks | 7400 | High | High |
|  |  | HR T-cell without Nelarabine + HR blocks | 4200 | Low | High |
|  |  | HR T-cell with Nelarabine single | 1000 | Low | Low |
|  |  | HR T-cell with Nelarabine single + HR blocks | 3200 | Low | Low |
|  |  | HR T-cell with Nelarabine addition | 2000 | Low | Low |
|  |  | HR T-cell with Nelarabine addition + HR blocks | 4200 | Low | High |
|  |  | DS-SR | 1000 | Low | Low |
|  |  | DS-IR, DS-HR | 3000 | Low | Low |
| LCH (Langerhans Cell | LCH IV | Stratum 1 group 1 (MS-LCH) arm A / B / C / D | 0 | Low | Low |
| Histiocytosis) |  | Stratum 1 group 2 (SS-LCH) | 0 | Low | Low |
|  |  | Stratum 2 | 0 | Low | Low |
|  |  | Stratum V without clinical neurodegeneration | 0 | Low | Low |
|  |  | Stratum V with clinical neurodegeneration | 0 | Low | Low |
| Hodgkin lymphoma | EuroNet-PHL-C2 | TL1 | 1000 | Low | Low |
|  |  | TL2 | 2000 | Low | Low |
|  |  | TL2 intensified | 2500 | Low | Low |
|  |  | TL3 | 4000 | Low | High |
|  |  | TL3 intensified | 5000 | Low | High |
| Non-B NHL (Non-Hodgkin Lymphoma) | Euro LB-02 | T-Cell LL stage I-II | 2000 | Low | Low |
|  |  | T-Cell LL stage III-IV | 3000 | Low | Low |
|  |  | Non-T-Cell LL stage I-II | 2000 | Low | Low |
|  |  | Non-T-Cell LL stage III-IV | 3000 | Low | Low |
| LBL (Lymphoblastic lymphoma) | EICNHL | Standard risk group, I ir II stages pB-LBL | 2000 | Low | Low |
|  |  | High risk group, III and IV stages pB-LBL ir T-LBL (all stages) | 3000 | Low | Low |
| B-NHL/B-ALL | NHL-BFM | R1 | 1976 | Low | Low |
| (B-cell Non-Hodgkin Lymphoma/ |  | R2 | 4352 | Low | High |
| acute lymphoblastic leukemia) |  | R3-R4-CNS- | 4352 | Low | High |
|  |  | R4-CNS+ | 4352 | Low | High |
|  | EICNHL | A | 3000 | Low | Low |
|  |  | B I | 3300 | Low | Low |
|  |  | B II (High risk) | 3300 | Low | Low |
|  |  | C1 | 5800 | Low | High |
|  |  | C3 | 5800 | Low | High |
|  | Inter-B-NHL ritux | Group B HR | 3300 | Low | Low |
|  |  | Group C1 | 5800 | Low | High |
|  |  | Group C3 | 5800 | Low | High |
|  |  | PMLBL | 4500 | Low | High |
| Anaplastic large | EICNHL | Isolated skin ALCL | 0 | Low | Low |
| cell lymphoma (ALCL) |  | Systemic ALCL: completely resected | 3352 | Low | High |
|  |  | Systemic ALCL: all other stages | 6328 | High | High |
|  |  | Patients with CNS+ | 4352 | Low | Low |
|  | ALCL | LR | 3352 | Low | Low |
|  |  | SR arm 1 SR arm 3, HR arm 1, HR arm 2, HR arm 3, HR arm 4 | 6328 | High | High |
| Acute myelod leukemia | Nopho DBH AML  2012 | SR | 0 | Low | Low |
|  |  | HR | 0+SCT | High | High |
| Acute promyelocytic | ICC APL 01 | SR MRD- / SR MRD+ / HR | 0 | Low | Low |
| leukemia | ICC APL 02 | SR, HR | 0 | Low | Low |
| Solid tumours | | | | | |
| Neuroblastoma | SIOPEN European Low and Intermediate Risk Neuroblastoma (LINES) | LR group 1 | 0 | Low | Low |
|  |  | LR group 2 2xVP/CARBO | 0 | Low | Low |
|  |  | LR group 2 2xVP/CARBO +2x CADO | 3000 | Low | Low |
|  |  | LR group 3 4x VP/CARBO | 0 | Low | Low |
|  |  | LR group 3 2x VP/CARBO + 2x CADO | 3000 | Low | Low |
|  |  | LR group 4 | 0 | Low | Low |
|  |  | LR group 5 2xVP/CARBO | 0 | Low | Low |
|  |  | LR group 5 2xVP/CARBO +2x CADO | 3000 | Low | Low |
|  |  | LR group 6 4x VP/CARBO | 0 | Low | Low |
|  |  | LR group 6 2x VP/CARBO + 2x CADO | 3000 | Low | Low |
|  |  | IR group 7 4x VP/CARBO | 0 | Low | Low |
|  |  | IR group 7 2x VP/CARBO + 2x CADO | 3000 | Low | Low |
|  |  | IR group 8 2x VP/CARBO + 2x CADO + 1xVP/CARBO + 1xCADO | 4500 | Low | High |
|  |  | IR group 8 2x VP/CARBO + 2x CADO + 2xCADO | 6000 | High | High |
|  |  | IR group 9 2x VP/CARBO + 2x CADO + 1xVP/CARBO + 1xCADO | 4500 | Low | High |
|  |  | IR group 10 4x VP/CARBO | 0 | Low | Low |
|  |  | IR group 10 2x VP/CARBO + 2xCADO | 3000 | Low | Low |
|  |  | IR group 10 2x VP/CARBO + 4x CADO | 6000 | High | High |
|  |  | IR group 10 4x VP/CARBO + 4x CADO | 6000 | High | High |
|  | HR-NBL-1.8/SIOPEN | BuMel any weight | >12000 | High | High |
| Ewing | Ewing 2008 | R1 female | 25176 | High | High |
|  |  | R3 | 25176 | High | High |
|  |  | R3 + TreoMel or BuMel | 30776 | High | High |
|  | Ewing 2012 | Arm A R2 VAC | 25140 | High | High |
|  |  | BuMel any weight | >23000 | High | High |
|  |  | Arm A R2 VAI | 24888 | High | High |
|  |  | Arm B R2 IEVC | 23772 | High | High |
|  |  | Arm B R2 BuMel any weight | >23000 | High | High |
| Osteosarcoma | EURAMOS 1 | MAP | 0 | Low | Low |
|  |  | MAPIE | 14640 | High | High |
| Renal tumours | UMBRELLA 2016/SIOP 2001 | AV + AVD, AV + AV1, AV + AV2 | 0 | Low | Low |
|  |  | AV + HR | 8100 | High | High |
| Rhabdoid tumors | EpSSG NRSTS 2005 | Cyclophosphamide | 17000 | High | High |
| of the kidney | EURHAB <18 mo | 3x DOX, 3x ICE, 3xVCA | 8892 | High | High |
| (RTK) or of soft | EURHAB <18 mo HD | 2x DOX, 2x ICE, 2x VCA + CARBO Thiotepa | 50928 | High | High |
| tissue (MRT) | EURHAB >18 mo | 3x DOX, 3x ICE, 3xVCA + RT | 8892 | High | High |
|  | EURHAB >18 mo HD | 2x DOX, 2x ICE, 2x VCA + CARBO Thiotepa + RT | 50928 | High | High |
| NRSTS* | EpSSG NRSTS 2005 | 3x ifosfamide | 6588 | High | High |
| (Non-Rhabdomyosarcoma Soft |  | 4x ifosfamide | 8784 | High | High |
| Tissue Sarcoma) |  | 5x ifosfamide | 10980 | High | High |
|  |  | 6x ifosfamide | 13176 | High | High |
| Soft tissue sarcomas | EpSSG RMS 2005 | LR subgroup A | 0 | Low | Low |
|  |  | SR subgroup B | 5800 | Low | High |
|  |  | SR subgroup C (9x Ifosfamide) | 13176 | High | High |
|  |  | SR subgroup C (5xIfosfamide) | 7320 | High | High |
|  |  | SR subgroup D (9x Ifosfamide) | 13176 | High | High |
|  |  | HR ir group A + group C | 13176 | High | High |
|  |  | HR ir group A + group D | 17376 | High | High |
|  |  | HR ir group B + group C | 13176 | High | High |
|  |  | HR ir group B + group D, VHR | 17376 | High | High |
|  | CWS-guidance-2014 | RMS Low risk subgroup A | 0 | Low | Low |
|  |  | RMS standard risk subgroup B | 5856 | Low | High |
|  |  | RMS standard risk subgroup C without RT | 13176 | High | High |
|  |  | RMS standard risk subgroup C with RT | 7320 | High | High |
|  |  | RMS standard risk subgroup D | 13176 | High | High |
|  |  | RMS High risk subgroups E, F, G | 13176 | High | High |
|  |  | RMS very High risk subgroup H | 13176 | High | High |
|  |  | “RMS-like” (SySa, STET (EES, pNET), UDS) | 13176 | High | High |
|  |  | “Non-RMS-like” (NRTS) Low risk | 0 | Low | Low |
|  |  | “Non-RMS-like” (NRTS) Intermediate risk | 0 | Low | Low |
|  |  | “Non-RMS-like” (NRTS) High risk | 13176 | High | High |
|  |  | Metastatic soft tissue tumours | 13176 | High | High |
|  |  | CWS-proposal for angiosarcoma | 6000 | High | High |
| Germ cell tumours | SIOP CNS GCT II | CCLG | 4392 | Low | High |
|  |  | NGGCT | 7320 | High | High |
|  |  | 2xPEI+2X HD-PEI (High risk non-germinoma) | 8540 | High | High |
|  | PEB+JEB blocks | Low risk | 0 | Low | Low |
|  |  | Intermediate risk | 0 (cis/carboplatin) | Low | Low |
|  |  | High risk | 0 (cis/carboplatin) | Low | Low |
| Hepatocellular | PHITT | Group A1 very Low risk HB | 0 | Low | Low |
| carcinoma |  | Group A2 very Low risk HB | 0 (cisplatin) | Low | Low |
|  |  | Group B1 Low risk HB / B2 | 0 (cisplatin) | Low | Low |
|  |  | Group C Intermediate risk SIOPEL3HR / C5VD/ CDDP-M | 0 (cisplatin) | Low | Low |
|  |  | Group D1 High risk HB SIOPEL4, D2 High risk HB CDCE, CDVI | 0 (cis/carboplatin) | Low | Low |
|  |  | Group E1 resected HCC | 0 | Low | Low |
|  |  | Group E2 resected HCC PLADO | 0 (cisplatin) | Low | Low |
|  |  | Group F not resected/metastatic PLADO sorafenib, GEMOX | 0 (cisplatin) | Low | Low |
| Brain tumours | | | | | |
| Optic glioma | SIOP LGG 2004 | Vincristine, carboplatin, etoposide (if allergy: cyclo) | 0 | Low | Low |
| Intradural-extramedulary tumor | HIT-MED + SCT |  | 49500 | High | High |
|  |  |  |  |  |  |
| Medulloblastoma | PNET 5 | MB-SR / MB-WNT-HR(>16years) | 17600 | High | High |
|  |  | MB-WNT-HR (<16years) | 13200 | High | High |
|  |  | MB-SHH-TP53: no alkylating agents | 0 | Low | Low |
|  | SR ACNS0331 | Cyclophosphamide, lomustine | 13200 | High | High |
|  | HR ACNS0332 | cyclophosphamide | 12000 | High | High |
|  | HIT-MED Guidance | 3x SKK | 7200 | High | High |
|  |  | 3x SKK+ 2x mSKK | 12000 | High | High |
|  |  | 3x intensified induction + 1^st^ HDCT + 2^nd^ HDCT + 6x maintenance | 4500+intensified induction | High | High |
|  |  | RT + 8x maintenance | 0 (cisplatin) | Low | Low |
|  |  | 2x SKK + RT + 4x maintenance | 4800 | Low | High |
| AT/RT (Atypical teratoid/rhabdoid tumors) | EURHAB <18 mo | 3x DOX, 3x ICE, 3xVCA | 8892 | High | High |
|  | EURHAB <18 mo HD | 2x DOX, 2x ICE, 2x VCA + CARBO Thiotepa | 50928 | High | High |
|  | EURHAB >18 mo | 3x DOX, 3x ICE, 3xVCA + RT | 8892 | High | High |
|  | EURHAB >18 mo HD | 2x DOX, 2x ICE, 2x VCA + CARBO Thiotepa + RT | 50928 | High | High |
| Ependymoma | HIT-MED Guidance | 3x SKK+2x mSKK | 12000 | High | High |
|  |  | Local RT | 0 | Low | Low |
|  |  | 2x mSKK + RT | 4800 | Low | High |
| Pineoblastoma | HIT-MED Guidance | CARBO/ETO-96h induction + 1st HDCT + 2nd HDCT + 6x maintenance) | 4500 | Low | High |
|  |  | RT + 8x maintenance | 0 (cisplatin) | Low | Low |
|  |  | 2x SKK + RT + 4x maintenance | 4800 | Low | High |
| High grade glioma, pons glioma | ACNS0126 | Temosolamide | 0 | Unknown | Unknown |
| HGG (High grade glioma) | Infant HGG 2013/HIT SKK | Elements IIs IIIs/1 IIIs/2 IVs | 7200 | High | High |

The allocated risk is based on the recently published IGHG guidelines (1,2). The risk allocation of patients with renal tumors is postponed until after surgery, when the definitive treatment including radiotherapy dose is known. For patients with ALL and NHL risk allocation is postponed to the moment of reaching complete remission (CR) or a treatment arm allocation. Some patients with large abdominal tumors at a high risk for infertility will be counseled at a later moment due to the desirability of abdominal surgery with a large abdominal tumor in situ.

* - Amended version of the infertililty risk stratification tool (triage) published by Princess Máxima Center (1).

** - CED (mg/m^2^) = 1.0 (cumulative cyclophosphamide dose, mg/m^2^) + 0.244 (cumulative ifosfamide dose, mg/m2) + 0.857 (cumulative procarbazine dose, mg/m^2^) + 14.286 (cumulative chlorambucil dose, mg/m^2^) + 15.0 (cumulative BCNU dose, mg/m^2^) + 16.0 (cumulative CCNU dose, mg/m^2^) + 40 (cumulative melphalan dose, mg/m^2^) + 50 (cumulative thiotepa dose, mg/m^2^) + 100 (cumulative nitrogen mustard dose, mg/m^2^) + 8.823 (cumulative busulfan dose, mg/m^2^).

*** - High risk of gonadal damage for females if CED ≥6000 mg/m^2^, ovarian radiotherapy (RT), allo-SCT – oocytes cryopreservation (OC) is strongly recommended, ovarian tissue cryopreservation (OTC) is moderately recommended (2)).

If low-dose alkylating agents (CED <6000 mg/m^2^), cranial RT, unilateral oophorectomy – OC is moderately recommended (2).

**** - High risk of gonadal damage for males if CED ≥4000 mg/m^2^, testicular RT, allo-SCT – sperm cryopreservation is strongly recommended (3).

If low-dose alkylating agents (CED <4000 mg/m^2^), cisplatin, orchiectomy, cranial RT – sperm cryopreservation is strongly recommended (3).

**References**

1. Perk MEM van der, Kooi ALLF van der, Wetering MD van de, IJgosse IM, Broeder E van D den, Broer SL, et al. Oncofertility care for newly diagnosed girls with cancer in a national pediatric oncology setting, the first full year experience from the Princess Máxima Center, the PEARL study. PLOS ONE. 2021 Mar 5;16(3):e0246344.

2. Mulder RL, Font-Gonzalez A, Hudson MM, Santen HM van, Loeffen EAH, Burns KC, et al. Fertility preservation for female patients with childhood, adolescent, and young adult cancer: recommendations from the PanCareLIFE Consortium and the International Late Effects of Childhood Cancer Guideline Harmonization Group. The Lancet Oncology. 2021 Feb 1;22(2):e45–56.

3. Mulder RL, Font-Gonzalez A, Green DM, Loeffen EAH, Hudson MM, Loonen J, et al. Fertility preservation for male patients with childhood, adolescent, and young adult cancer: recommendations from the PanCareLIFE Consortium and the International Late Effects of Childhood Cancer Guideline Harmonization Group. The Lancet Oncology. 2021 Feb 1;22(2):e57–67.
